# Naturalizing laboratory mice by housing in a farmyard-type habitat confers protection against colorectal carcinogenesis

**DOI:** 10.1080/19490976.2021.1993581

**Published:** 2021-11-09

**Authors:** Henriette Arnesen, Thomas C. A. Hitch, Christina Steppeler, Mette Helen Bjørge Müller, Linn Emilie Knutsen, Gjermund Gunnes, Inga Leena Angell, Ida Ormaasen, Knut Rudi, Jan Erik Paulsen, Thomas Clavel, Harald Carlsen, Preben Boysen

**Affiliations:** a Department of Preclinical Sciences and Pathology, Faculty of Veterinary Medicine, Norwegian University of Life Sciences (NMBU), Aas, Norway; b Faculty of Chemistry, Biotechnology and Food Science, Norwegian University of Life Sciences (NMBU), Norway; c Functional Microbiome Research Group, Institute of Medical Microbiology, University Hospital of RWTH Aachen, Aachen, Germany; d Department of Paraclinical Sciences, Faculty of Veterinary Medicine, Norwegian University of Life Sciences (NMBU), Aas, Oslo, Norway

**Keywords:** Gut microbiota, feralized mice, colorectal cancer, farmyard-like habitat, animal model, naturalized mice, short-chain fatty acids, immunity

## Abstract

Living in a farm environment in proximity to animals is associated with reduced risk of developing allergies and asthma, and has been suggested to protect against other diseases, such as inflammatory bowel disease and cancer. Despite epidemiological evidence, experimental disease models that recapitulate such environments are needed to understand the underlying mechanisms. In this study, we show that feralizing conventional inbred mice by continuous exposure to a livestock farmyard-type environment conferred protection toward colorectal carcinogenesis. Two independent experimental approaches for colorectal cancer induction were used; spontaneous (Apc Min/+ mice on an A/J background) or chemical (AOM/DSS). In contrast to conventionally reared laboratory mice, the feralized mouse gut microbiota structure remained stable and resistant to mutagen- and colitis-induced neoplasia. Moreover, the feralized mice exhibited signs of a more mature immunophenotype, indicated by increased expression of NK and T-cell maturation markers, and a more potent IFN-γ response to stimuli. In our study, hygienically born and raised mice subsequently feralized post-weaning were protected to a similar level as life-long exposed mice, although the greatest effect was seen upon neonatal exposure. Collectively, we show protective implications of a farmyard-type environment on colorectal cancer development and demonstrate the utility of a novel animal modeling approach that recapitulates realistic disease responses in a naturalized mammal.

## Graphical abstract

**Figure:**
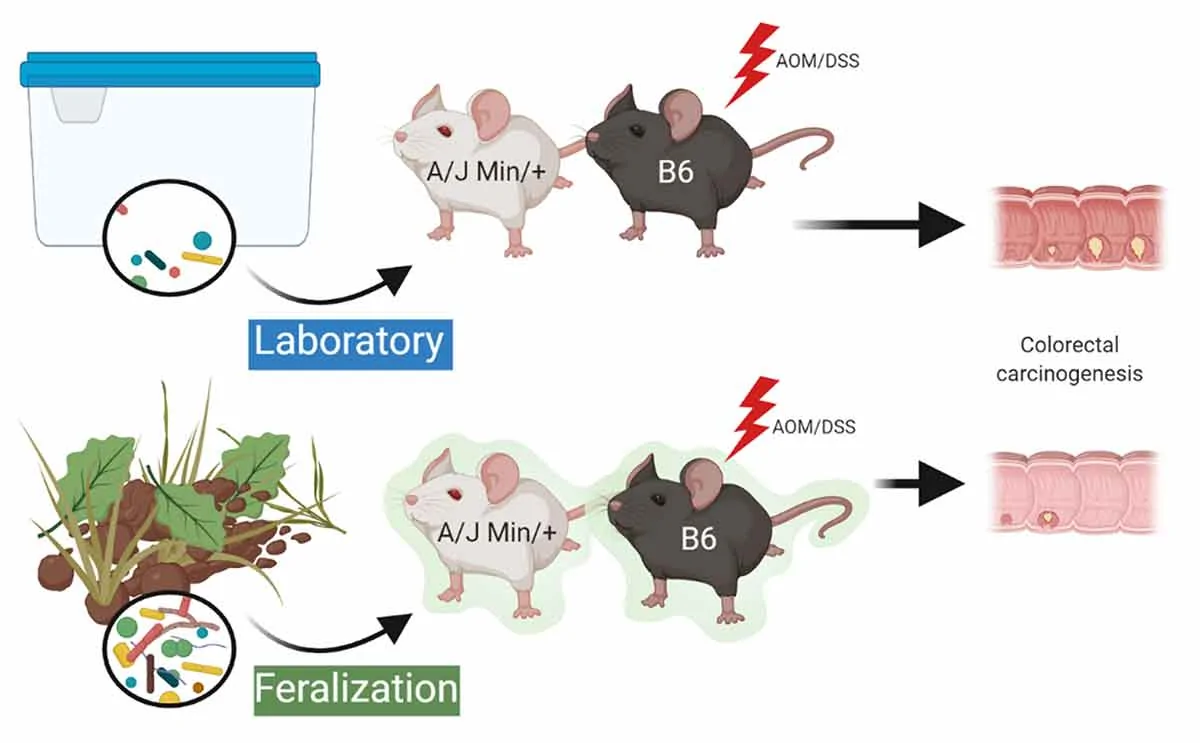

## Introduction

The mammalian gut hosts a complex and diverse ecosystem, which has co-evolved with the host to form a symbiotic relationship fundamental for host fitness. The gut microbiota has been shown to shape host immunity during development, ensuring adequate defense toward potentially harmful pathogens and tolerance to commensal species.^1^^, 2^ The gut microbiota is acquired and influenced by both vertical and horizontal transmission from maternal and environmental sources.^3,4^ Epidemiological studies have demonstrated that children exposed to high microbial biodiversity environments harbor different microbiomes and enhanced immune regulation than urban children,^5^ and are less susceptible to diseases, such as asthma and allergies.^6–8^ Moreover, farmers have reduced risk of certain types of cancer.^9^ A connection between decreased environmental biodiversity accompanying an urban living and increased risk for inflammatory bowel diseases (IBDs) has also been suggested.^10,11^ Given that individuals with IBDs have substantially increased risk for colorectal cancer (CRC),^12,13^ it can be hypothesized that exposure to environmental microbes and previous infections may also influence the risk for CRC.

CRC is the second most diagnosed cancer in women, and the third most common malignancy in men worldwide.^14,15^ A minority of CRC cases are attributed to hereditary factors, such as germline mutations in susceptibility genes, while most CRCs arise sporadically and can be influenced by various environmental components, with gut microbiota as a unifying factor.^16,17^ Both genetic and inducible CRC mouse models are commonly used to study the multifaced mechanisms behind CRC. Apc Min/+ mice harbor a mutant allele of the adenomatous polyposis coli (*Apc*) gene and spontaneously develop adenomatous polyps.^18,19^ CRC can also be induced chemically by e.g. a combinatory treatment of the pro-carcinogen azoxymethane (AOM) and the inflammatory agent dextran sodium sulfate (DSS). The AOM/DSS model is considered robust and has emerged to become one of the most frequently used models to study inflammation-associated CRC. In this model, carcinogenesis is induced by AOM metabolism to alkylating species generating DNA mutations, while the subsequent colonic epithelial damage inflicted by DSS promotes the carcinogenic process.^18^

To decipher the role of gut microbiota in health and disease, engraftment of minimal or specific microbial communities in gnotobiotic and germ-free mice have been widely employed, allowing for controlled composition of gut commensals.^20^ However, increased awareness of the tandem function of gut microbes and host immune system has engendered concerns over the potential bias introduced by hygienic housing on the microbiota and its downstream effect on disease modeling in mice.^21,22^ Moreover, while lab mice are known to have less microbiota variation than wild mice, the between-lab/vendor variability has been shown to alter the outcomes in disease models.^23,24^ In essence, a growing body of research highlights that conventional lab mice are too far removed from their natural, usually microbially rich, habitat to accurately reflect the immunological responses of free-living mammals and humans.^25–32^ In recent years, several approaches to study the implications of naturalized lab mice have been presented.^26,27,29,30,33^ These studies show that naturalization of lab mice result in clear shifts in gut microbiota and more mature immunophenotypes, as well as protection against various diseases, including that a wild mouse microbiota mitigate CRC outcome.^29^

We have established a simulated natural indoor housing facility in which lab mice could be feralized in a farmyard-type setting with feral mice cohabitants, leading to distinct changes in immune parameters and gut microbiota.^34^ In the current study, we employed this feralization model and found that a farmyard-type habitat itself, in the absence of feral mice, effectively dampened CRC development in the AOM/DSS as well as the A/J Min/+ models of CRC. We characterized the gut microbiota and immune parameters as potential drivers of differential disease outcomes in the feralized mice.

## Results

### Genetically susceptible Min/+ mice feralized in a naturalistic environment showed reduced rate of colonic lesion formation

**Figure 1.**
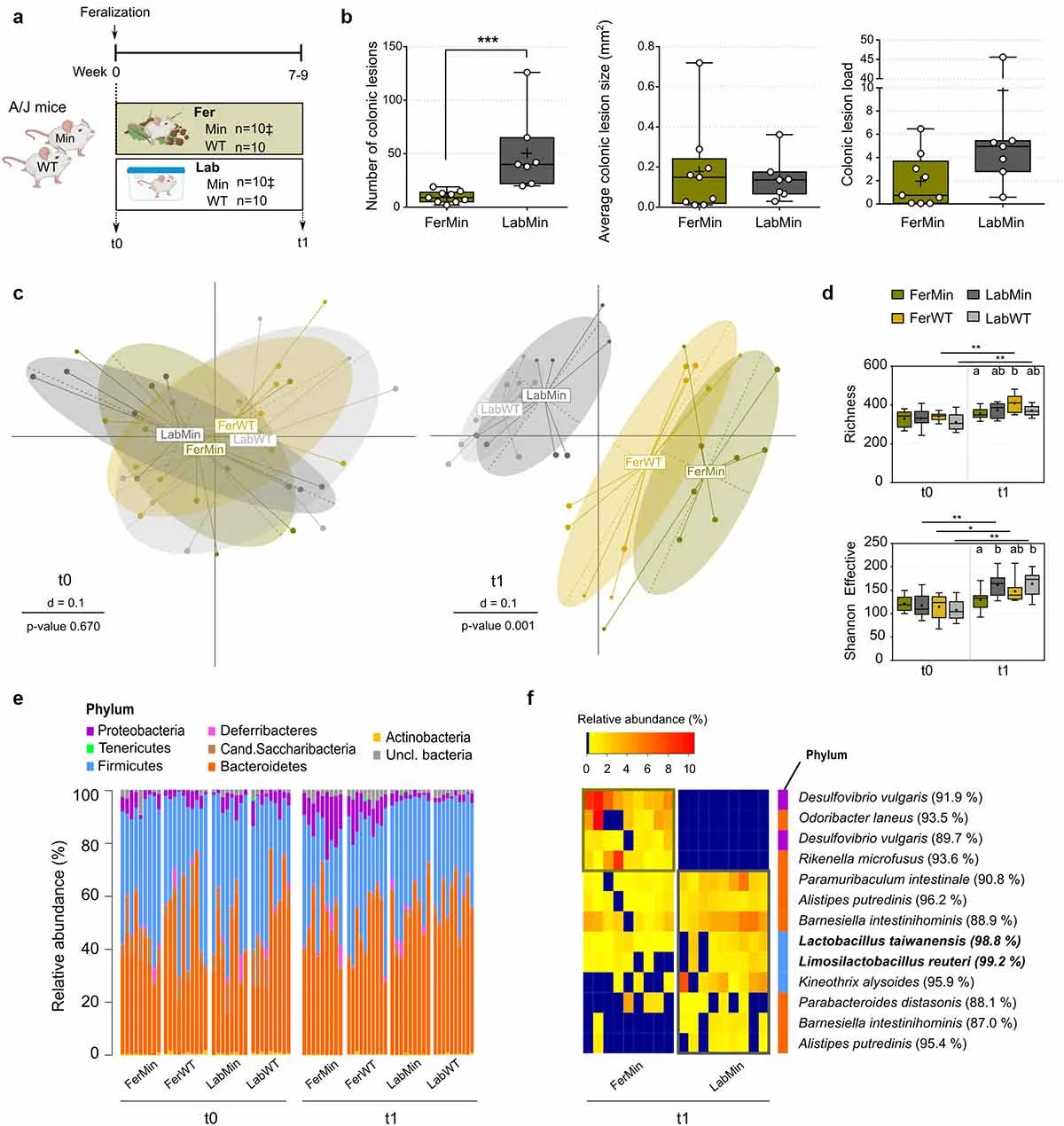
Feralization of A/J Min/+ mice led to diminished spontaneous colonic lesion formation, accompanied by altered microbiota profile. (a) Scheme of the experimental setup showing timeline and grouping. 6-8-week-old female mice were enrolled. Samples were collected at baseline (t0; week 0) and endpoint (t1; week 7–9). ‡ one mouse deceased before endpoint and were consequently excluded from endpoint analyses. (b) Assessment of colonic lesions in Fer and Lab A/J Min/+ (FerMin and LabMin) mice at endpoint. The occurrence of lesions is presented as total number, mean size (mm^2^), and load (total mm^2^). Box plots show median (line), mean (+), IQR (box) and minimum to maximum (whiskers). Asterisks designate significant (***p ≤ 0.001) differences between the groups determined by Mann-Whitney tests. (c) Multi-dimensional scaling (MDS) plot of fecal microbiota profiles (generalized UniFrac distances) for Fer and Lab A/J Min/+ (FerMin, LabMin) and WT (FerWT, LabWT) mice at baseline (t0) and endpoint (t1). Significance of separation was determined by PERMANOVA. d = distance scale. (d) Observed number of OTUs (Richness) and Shannon Effective counts for all groups. Box plots show median (line), mean (+), IQR (box) and minimum to maximum (whiskers). Asterisks designate significant over-time differences determined by Wilcoxon Signed Rank Sum tests, whilst letters designate significant (p ≤ 0.05) differences between groups at each timepoint determined by Kruskal-Wallis tests followed by Wilcoxon Rank Sum tests. The Benjamini–Hochberg method was used to correct for multiple testing. (e) Taxonomic binning at phylum level, presented as relative abundance for Fer and Lab A/J Min/+ (FerMin, LabMin) and WT (FerWT, LabWT) mice at baseline (t0) and endpoint (t1). (f) Heatmap of relative abundance of specific OTUs enriched in Fer and Lab A/J Min/+ (FerMin, LabMin) mice at endpoint (t1). The occurrence of OTUs for which the relative abundance or prevalence differed significantly between the groups (determined by Wilcoxon Rank Sum tests and Fisher’s exact tests, respectively) are plotted. Blue color indicates the OTUs were absent or below cutoffs for analyses. The bacterial species with a valid name closest to the corresponding OTUs is indicated along with its sequence similarity; those OTUs identifiable at the species level (≥97% similarity) are written in bold letters. Phyla to which the OTUs belong are designated with colored squares as specified in E. Frames indicate significant increased abundance or prevalence in FerMin (brown) and LabMin (gray). See also **Figures S1 and S2A.**

Young adult male A/J Min/+ and A/J wild-type (WT) mice were either feralized (Fer) in a simulated natural environment or housed in clean conventional cages (Lab) for 7–9 weeks (Figure 1a). Changes in bodyweight were similar in all A/J Min/+ mice independent of the housing conditions (**Figure S1A**). In the feralized A/J Min/+ mice (FerMin), the number of observed colonic lesions were significantly reduced compared to the Lab mice (LabMin) (Figure 1b). The mean lesion size or load (sum of lesion area) between the groups were not significantly different, reflecting that the increased number of lesions in LabMin mice were small-sized lesions. The small intestines (SI) were also examined and scored, as A/J Min/+ mice have been previously shown to also develop SI lesions.^19^ We observed a substantial number of SI lesions in both FerMin and LabMin mice. However, no significant differences in number, size or load between the two groups were observed (**Figure S1B**).

**Table 1.** Histopathological classification of colonic lesions. Presented are numbers of individual mice in which at least one lesion within the class was detected, and mean numbers of lesions detected within each class and in total in the given groups. SDs are presented in brackets. *p ≤ 0.05, significant difference between groups (determined by Kruskal–Wallis test followed by Dunn’s multiple comparisons tests)

	FerE+		FerL+		Lab+	
Hyperplasia	5/6	1.33 (1.03)	6/6	2.17 (1.17)	6/6	3.83 (3.06)
Adenoma	6/6	1.50 (0.84)	5/6	4.50 (3.62)	6/6	5.00 (3.46)
Carcinoma	0/6	-	0/6	-	0/6	-
Total	6/6	2.83 (1.33)*	6/6	6.67 (3.67)	6/6	8.83 (5.04)*

### Feralization of A/J Min/+ mice led to altered gut microbiota profile with enrichment of Proteobacteria

To characterize the influence of feralization on the gut microbiota, stool samples were collected for high-throughput 16S rRNA gene (V3-V4) amplicon analysis. This resulted in 1,002,551 high-quality and chimera-checked sequences (6,780 to 29,032 per sample), which represented a total of 670 OTUs. Sequencing depth was evaluated by rarefaction curves to confirm each sample's suitability for further analysis (**Figure S2A**).

Before separating the A/J Min/+ or A/J WT mice into Fer or Lab conditions, gut microbiota profiles were similar in all groups, but separated substantially following their introduction into the different housing conditions, confirming that the two environments differentially influenced gut microbiota structure (Figure 1c). *Alpha*-diversity measures showed that the number of detected molecular species (OTUs) (richness) and Shannon effective counts were similar in all groups at the starting point. At endpoint, the FerMin mice had significantly lower richness compared to the feralized WT mice (FerWT) (*p* = .012), and significantly lower Shannon effective compared to both of the conventionally housed groups (i.e. LabMin and corresponding WT mice; LabWT) (*p* = .043 and *p* = .024, respectively) (Figure 1d).

The changes in gut microbiota conferred by feralization were apparent at the taxonomic rank of phylum, where we detected significantly higher relative abundances of Proteobacteria in both FerMin and FerWT mice than in the LabMin and LabWT (all comparisons p ≤ 0.001) (Figure 1e). No differences at the phylum level were detected at baseline, indicating that the Proteobacteria colonization was a result of the environmental influence rather than genotypic differences or disease state.

To further characterize the differences in the Fer and Lab gut microbiotas at endpoint, we conducted analysis at the level of specific OTUs (figure 1f). Two OTUs with closest sequence similarity to a member of the Proteobacteria, *Desulfovibrio vulgaris*, were detected in nearly all (9/9 and 8/9) FerMin mice and were completely absent in LabMin mice. In contrast, OTUs showing closest sequence similarities to members of the phylum Firmicutes, including two species assigned to *Lactobacillus* and *Limosilactobacillus* were enriched in the LabMin mice. Similar results were seen for the FerWT and LabWT mice, indicating that environmental influence rather than disease state was a major driver for the microbial differences (**Figure S1C**).

### Feralization alleviated mutagen- and colitis-induced carcinogenesis in B6 mice

**Figure 2.**
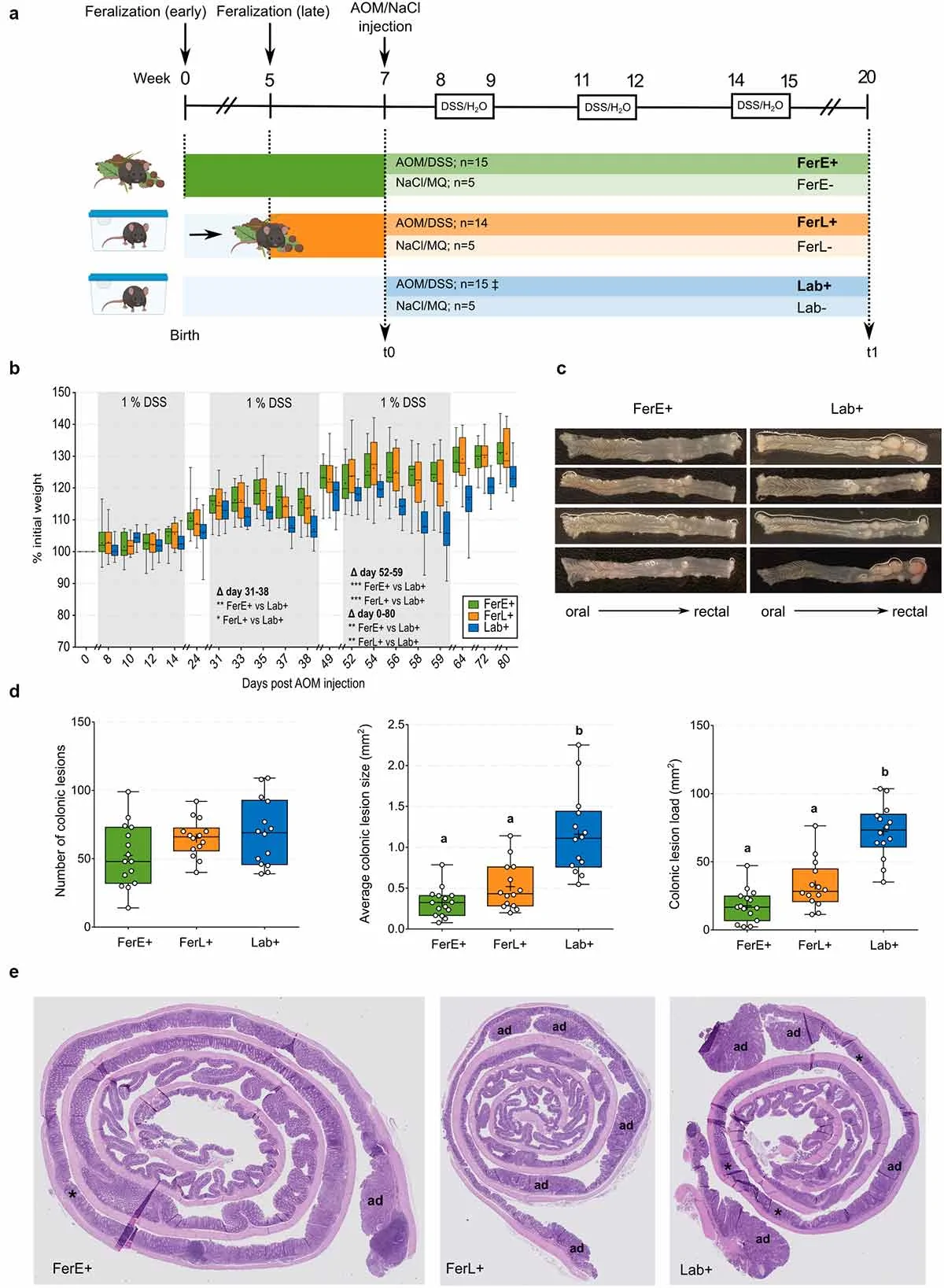
Feralization conferred protection toward mutagen- and colitis-induced carcinogenesis in B6 mice. (a) Chart showing timeline of the AOM/DSS trial and the grouping of animals. Timeline start at birth (week 0). Animals born in the mouse pens by feralized mothers compose the early feralized (FerE) groups, whilst animals born in the laboratory by non-feralized mothers compose the Lab groups. At week 5, feralization of a subset of Lab animals gave rise to the late feralized (FerL) groups. At week 7, CRC was induced by AOM injection followed by DSS administration. Control groups were given NaCl injection and H_2_O. Samples were collected prior to AOM or NaCl injection (t0; week 7) and at trial termination (t1; week 20). ‡ one mouse deceased before endpoint and were consequently excluded from endpoint and over-time analyses. (b) Bodyweight curves for AOM/DSS-treated animals, presented as per cent of initial body weight. Box plots show median (line), mean (+), IQR (box) and minimum to maximum (whiskers). Significant changes in bodyweight from first to last day of the trial (0 to 80), and first to last day of each cycle (8–14, 31–38, 51–59), were determined using repeated measures ANOVA with Tukey multiple comparison tests and indicated in the figure. *p ≤ 0.05, **p ≤ 0.01, ***p ≤ 0.001. (c) Representative macroscopic pictures of colons from four AOM/DSS-treated FerE (left) and Lab (right) mice. Colons were cut longitudinally and are presented with luminal side facing up, from oral to rectal end, as indicated. Pictures are taken at termination (day 80), prior to formalin fixation. (d) The occurrence of colonic lesions following AOM/DSS treatment measured by total number of colonic lesions, mean size of lesions, and lesion load. Box plots show median (line), mean (+), IQR (box) and minimum to maximum (whiskers). Significance was determined using Kruskal Wallis followed by Dunn’s multiple comparison test. Different letters designate statistical significance with alpha level 0.05. (e) Images of H&E-stained colonic sections from FerE+, LabF+ and Lab+ groups. Presented are the most severely diagnosed sections per group. Ad, adenoma; *, hyperplasia. See also Table 1, **Figures S3 and S8.**

We proceeded to employ a chemical induction (AOM/DSS) model of CRC using female C57BL/6 JRj (B6) mice to evaluate the influence of feralization independent of genetic susceptibility. The B6 mice were separated into Fer and Lab groups prior to the chemical induction (Figure 2a). To investigate the influence of early-life versus later-in-life colonization, we included second generation feralized animals born by feralized mothers, which had been feralized from birth onwards (Feralized Early; FerE), and animals born in the lab setting by lab mothers and feralized after weaning, at 5 weeks of age (Feralized Late; FerL). The groups were administered AOM/DSS (+) or control treatment (-).

The AOM/DSS-treated Lab+ mice lost significantly more weight than both FerE+ and FerL+ mice during the second and third cycles of DSS treatment, as well as over the whole trial (Figure 2b). Colonic lesions formed from the AOM/DSS treatment were macroscopically different between FerE+ and Lab+ mice, with more prominent tumors in the latter group, as depicted in Figure 2c. The total number of lesions in AOM/DSS-treated mice detected by surface microscopy were, not different between Fer and Lab groups, yet the mean lesion size and load were significantly lower in FerE+ and FerL+ mice than in Lab+ mice (Figure 2d). Neither lesion numbers, size nor load were significantly different between FerE+ and FerL+ animals, although the latter group did show a phenotype in between FerE+ and Lab+ (Figure 2d).

Following lesion scoring by surface microscopy, histopathological assessment was conducted of the colons (Figure 2e**, Figure S8B**). Hyperplasia, adenomas, and carcinomas were classified and counted in the six colons with the highest mean lesion sizes in each group. The numbers of adenomas ranged from 1–3 in the FerE+, 0–10 in FerL+ and 2–11 in Lab+ groups, while the number of hyperplastic lesions ranged 0–3 in FerE+, 1–4 in FerL+ and 1–7 in Lab+. No carcinomas were diagnosed in any of the colons (Table 1). Statistical comparisons of the total numbers of lesions diagnosed by histopathology showed significant difference between the groups (*p* = .016) with pairwise comparisons revealing significantly lower numbers in FerE+ compared to Lab+ (*p* = .030) yet no significant differences between FerL and the other groups.

In the control-treated groups, we detected no bodyweight loss. The bodyweight of both FerE- and FerL-mice increased compared to Lab-mice, as indicated by significantly different weight change from first to last day of the trial (**Figure S3A**). As would be expected, hardly any colonic lesions were observed in the control-treated groups (**Figure S3B**).

Because an inverse relationship between physical exercise and CRC outcome in mice has been reported,^35,36^ we wished to assess whether the larger area of the naturalistic environment compared to the conventional lab cages could be responsible for the differences observed between feralized and lab mice. Thus, at week 7, five mice from both the FerE+ and FerL+ groups were placed into cages, along with environmental samples from their respective pens, to retain the environment, while otherwise treated with AOM/DSS (**Figure S3C**). Comparisons of bodyweight curves between the cage- and pen-housed Fer mice showed that FerE+ gained more weight than FerE^cage^+ over the whole trial period (**Figure S3D)**. The FerL^cage^+ group lost significantly more weight than the corresponding FerL+ group during second cycle of DSS treatment, but apart from this the bodyweight curves for the two groups were comparable during the trial (**Figure S3E**). However, the lesion assessment data for FerE^cage^+ and FerL^cage^+ matched the findings from FerE+ and FerL+ mice, and no significant effects of the cage housing were detected for any of the lesion measurements (**Figure S3F**). Fluid intake was similar in all groups (**Figure S3G**), confirming that the phenotypic features were not due to unequal DSS consumption.

Taken together, these findings demonstrate that feralization in a naturalistic environment confers traits that limit the impact of mutagen- and colitis-induced carcinogenesis. Feralization from birth and from later in life both mitigated CRC outcome, yet the protection was most pronounced in the early feralized group. The protective effect of feralization was shown to be largely independent of the enlarged space in the mouse pens.

### The gut microbiota of feralized B6 mice was distinct from that of laboratory mice and unaffected by mutagen- and colitis-induced carcinogenesis

Laboratory tests for common mouse pathogens were negative in fecal samples from mice representative for the Fer as well as the Lab groups (**Figure S4**). Likewise, standard examination (McMasters and immunofluorescent antibody testing for *Cryptosporidium* and *Giardia*) of mouse feces for parasites were negative. To unravel the influence of feralization on the gut microbiota structure in the chemical induction model, stool samples for all animals in the AOM/DSS trial were collected for high-throughput 16S rRNA gene amplicon sequencing. The analyzed 16S rRNA (V3-V4) amplicon dataset included 2,157,819 high-quality and chimera-checked sequences (8,266 to 26,531 per sample), which represented a total of 322 OTUs. Sequencing depth was evaluated by rarefaction curves to confirm the suitability of each sample for further analysis (**Figure S2B**).

**Figure 3.**
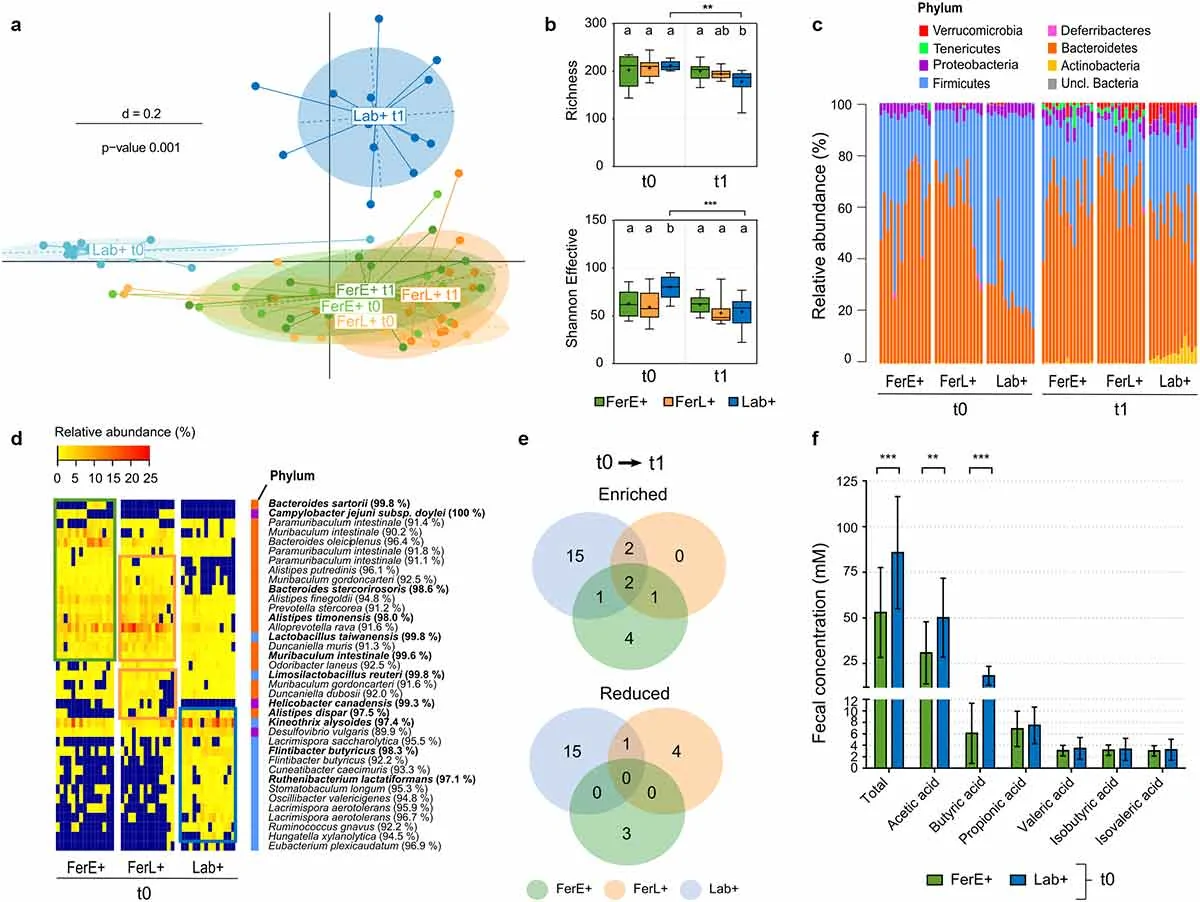
The gut microbiota of feralized and laboratory B6 mice significantly differed in composition and response to mutagen- and colitis-induced carcinogenesis. (a) Multi-dimensional scaling (MDS) plot of fecal microbiota profiles (generalized UniFrac distances) for AOM/DSS treated groups at baseline (t0) and endpoint (t1). Significance of separation was determined by PERMANOVA. d = distance scale. (b) Observed number of OTUs (Richness) and Shannon Effective counts for AOM/DSS treated groups. Box plots show median (line), mean (+), IQR (box) and minimum to maximum (whiskers). Asterisks designate significant (**p ≤ 0.01, ***p ≤ 0.001) over-time differences determined by Wilcoxon Signed Rank Sum tests, whilst letters designate significant (p ≤ 0.05) differences between groups at each timepoint determined by Kruskal-Wallis tests followed by pairwise Wilcoxon Rank Sum tests with Benjamini-Hochberg correction for multiple comparisons. (c) Taxonomic binning at the rank of phylum, presented as relative abundance for each individual. (d) Heatmap of relative abundance of specific OTUs enriched in Fer and Lab mice at baseline (t0). The occurrence of OTUs for which the relative abundance or prevalence differed significantly between the groups (determined by Kruskal-Wallis and Fisher’s exact test, respectively) are plotted. Blue color indicates the OTUs were absent or below cutoffs for analyses. The bacterial species with a valid name closest to the corresponding OTUs is indicated along with its sequence similarity; those OTUs identifiable at the species level (≥97% similarity) are written in bold letters. Phyla to which the OTUs belong are designated with colored squares as specified in c. Frames indicate significant increased relative abundance or prevalence in FerE (green), FerL (Orange) and Lab (blue) compared to one of the other groups determined by pairwise analyses (Wilcoxon Signed Rank Sum/Fisher’s Exact tests with Benjamini-Hochberg correction for multiple comparisons). (e) Venn diagrams of shared enriched and reduced OTUs among the three groups in response to AOM/DSS treatment. Significant over-time (t0-t1) differences within each group at the OTU-level was determined by Wilcoxon Signed Rank Sum and Fisher’s exact tests. Details are listed in Table S1. (f) Concentration of SCFAs in fecal samples obtained from Fer and Lab mice prior to AOM/DSS/control treatment, presented as mean with the standard deviation (SD) shown via the whiskers. Significance between groups was determined by Wilcoxon Rank Sum tests for each SCFA. **p ≤ 0.01, ***p ≤ 0.001. Lab+ n = 13, FerE+, n = 15. See also **Figure S2B, Figures S4-S6 and Table S1.**

*Beta*-diversity analysis identified significant clustering according to the environmental setting of AOM/DSS-treated animals (Figure 3a). The FerE+ and FerL+ groups formed distinct clusters from the Lab mice, both pre- (t0) and post-AOM/DSS treatment (t1). Pairwise comparisons confirmed that these observations were significant (**Figure S5A-C**). No significant difference was detected between FerE+ and FerL+ at baseline, demonstrating that the animals feralized at 5 weeks of age had approached a profile more similar to animals feralized from birth on than to Lab mice (**Figure S5A**). The richness was similar in the three groups at baseline, while effective Shannon counts were significantly lower in the FerE+ and FerL+ compared to Lab+ (both comparisons *p* = .003), suggesting a microbiota dominated by fewer dominant bacterial species (Figure 3b).

The difference in gut microbiota elicited by the feralization was evident in the relative abundance of taxa at the level of phyla (Figure 3c). Prior to AOM/DSS treatment, both FerE+ and FerL+ had a significantly higher relative abundance of Bacteroidetes and lower relative abundance of Firmicutes compared to Lab+ (all comparisons p ≤ 0.001). Moreover, Deferribacteres was only detected in the feralized mice. Analysis at the OTU level for timepoint 0 (t0) showed that the majority of molecular species enriched in the FerE+ and FerL+ mice are members of the Bacteroidetes, while OTUs belonging to Firmicutes were enriched in Lab+ mice (Figure 3d).

DSS treatment induces colitis accompanied by substantial changes in mice gut microbiota composition.^37,38^ Thus, we expected significant shifts in gut microbiota profiles in response to the AOM/DSS treatment. Surprisingly, Fer mice responded minimally to the AOM/DSS treatment, in contrast to the Lab mice (Figure 3a) (**Figure S5B**). *Alpha*-diversity measures remained unchanged in both FerE+ and FerL+ over time, while the Lab+ mice were characterized by a substantial reduction in both richness and Shannon effective counts (both p ≤ 0.001) (Figure 3b). A marked shift from Firmicutes to Bacteroidetes domination was observed for the Lab+ mice following the AOM/DSS treatment (Figure 3c). In contrast, the effect of AOM/DSS treatment on the dominating phyla of the Fer animals was modest, with only a minor yet significant decrease in Firmicutes in the FerL+ group. Among the less abundant phyla, Tenericutes was only detected in the Fer mice and bloomed after AOM/DSS treatment. Tenericutes was represented by a single OTU with closest sequence similarity to *Anaeroplasma bactoclasticum* (91.8%). All groups showed enrichment of Verrucomicrobia following AOM/DSS treatment, represented by a single OTU with closest sequence similarity to *Akkermansia muciniphila* (99.8%). This OTU was observed to be borderline more prevalent in Lab+ mice compared to FerE+ at endpoint (*p* = .051) (**Figure S5D**). Moreover, a bloom of Actinobacteria were observed in Lab mice after AOM/DSS treatment, largely due to one OTU with closest sequence similarity to *Bifidobacterium animalis* (99.8%) (**Figure S5D**). These results support that the Fer and Lab mice responded differently to the AOM/DSS treatment and suggests that the microbiota of the Fer mice was more resistant to treatment, compared to the Lab mice microbiota, which showed substantial changes (Figure 3e**, Table S1**).

We also characterized the gut microbiota profiles and composition of the control-treated groups (**Figure S6A-C**) and the cage-housed feralized groups (FerE^cage^+ and FerL^cage^+) (**Figure S6D-F**). Comparisons of AOM/DSS-treated and control-treated groups showed that the gut microbiota profiles of feralized mice clustered independently of treatment, while the Lab+ and Lab- were separate, particularly at endpoint (**Figure S6A**). The cage-housed Fer groups, FerE^cage^+ and FerL^cage^+, showed gut microbiota profiles overlapping those of FerE+ and FerL+ mice, respectively (**Figure S6D**). We found significant separation of gut microbiota profiles at endpoint yet pairwise comparisons showed no significant separation between FerL+ and FerL^cage^+ (*p* = .137) nor FerE+ and FerE^cage^+ (*p* = .072) groups. These data largely indicate that the farmyard-type environment rather than the enlarged space in the mouse pens influenced the gut microbiota profiles.

### The feralized gut microbiome is characterized by low fecal levels of SCFAs and relative abundance of short-chain fatty acid producers

To investigate possible cancer protective mechanisms, fecal concentrations of short-chain fatty acids (SCFAs) were measured. SCFAs are microbially derived molecules known to have immunomodulatory effects in the gut.^39,40^ A panel of SCFAs was analyzed in feces collected from FerE+ and Lab+ mice, before AOM/DSS treatment (t0; Figure 2a). In agreement with previous studies, we identified acetic acid as the dominant SCFA in our samples, followed by butyric acid and propionic acid.^41^ Notably, the FerE+ mice had significantly lower amounts of butyric and acetic acid, as well as total SCFAs, compared to the Lab+ mice (figure 3f). This was reflected in the baseline microbiota, where Lab+ mice showed higher relative abundances or prevalence of species in the Firmicutes phylum, specifically OTUs showing the closest species similarity to known butyrate-producing bacteria such as *Flintibacter butyricus*^42^ and *Kineothrix alysoides*^43^ (Figure 3d). However, through the course of AOM/DSS treatment, these OTUs were reduced in Lab+ mice (Figure 3e**, Table S1**).

### Immune cells of feralized B6 mice displayed a mature phenotype and demonstrated enhanced IFNγ T-cell response

To identify possible immunological factors likely to be involved in cancer protection, we conducted immunophenotyping of cells based on previous findings in feralized mice^34^ and of relevance in anti-tumor responses. Cells were harvested from spleen and mesenteric lymph nodes (mLNs) from all animals at endpoint (t1), and flow cytometry gating strategies are shown in **Figure S7**.

**Figure 4.**
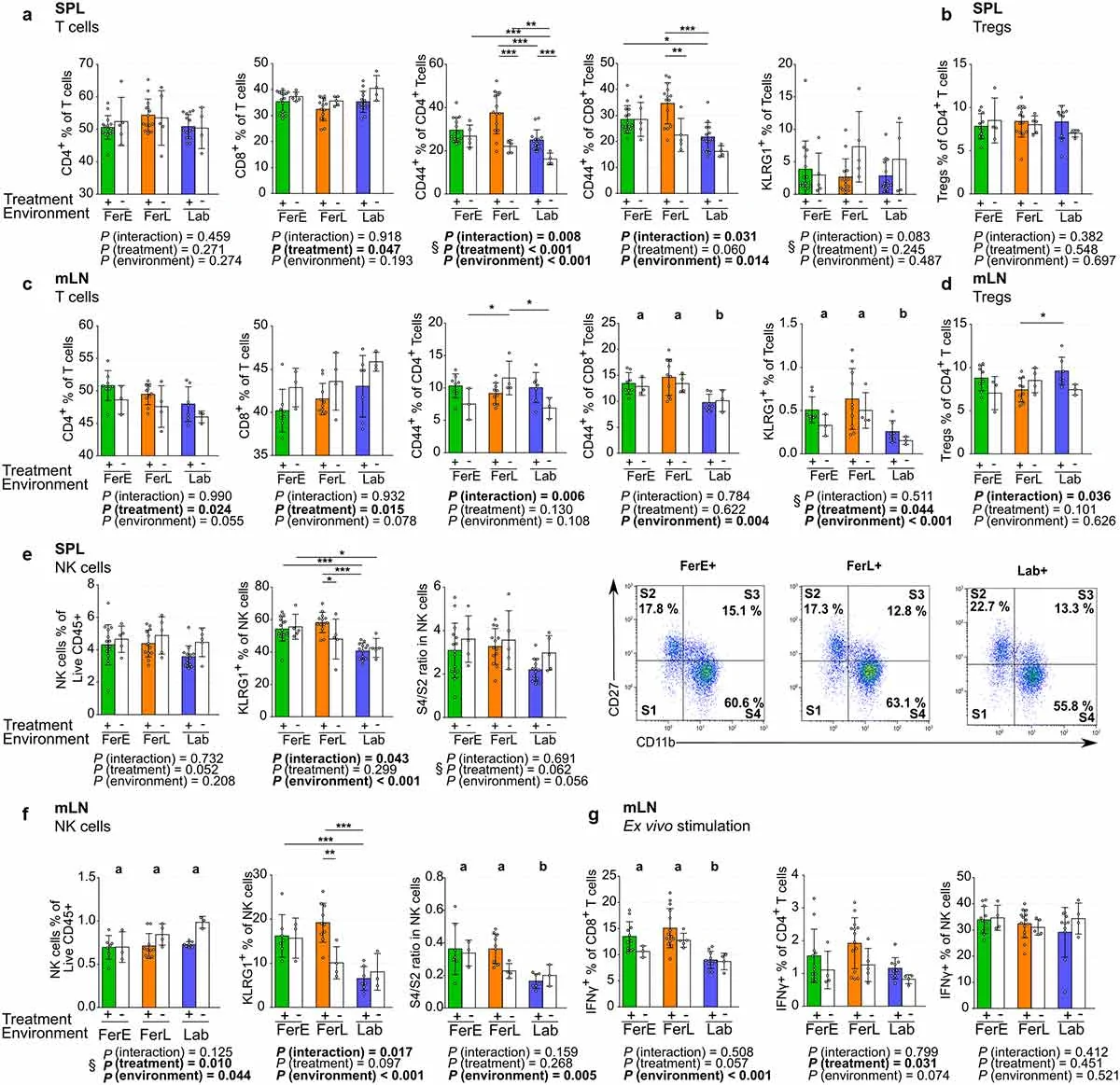
The feralized T and NK cells showed higher expression of maturation markers and increased IFNγ response to *ex vivo* stimuli. Phenotypic markers of (a) T-cells in spleen (SPL), (b) Tregs in spleen, (c) T-cells in mesenteric lymph nodes (mLN), (d) Tregs in mLN, (e) NK cells in SPL and (f) NK cells in mLN of Fer and Lab mice treated with AOM/DSS (+) or NaCl/H_2_O (-).In C, representative flow cytometric plots of maturation stages S1-S4 based on CD27 and CD11b expression are shown for the AOM/DSS treated FF, LabF and Lab groups. **(E)** Cells expressing IFNγ as % of CD4^+^ T-cells, CD8^+^ T-cells and NK cells. Cells were isolated from mLNs, cultured with PMA and ionomycin (for T-cell activation) or IL-2+ IL-13 (for NK cell activation) for 4 hours prior to immunophenotyping. All graphs are presented as mean, with the standard deviation (SD) shown via the whiskers. Statistical differences were determined by two-way ANOVA, with the *P* values for the main effects written out below each plot (significant results at alpha level 0.05 in bold letters). Different letters designate only statistical significance (p ≤ 0.05) between environments (FerE; FerL; Lab) determined by post hoc Tukey’s multiple comparison tests. Where interaction effects were detected, post hoc Bonferroni’s multiple comparison tests were conducted, and asterisks designate statistical significance (*p < .05, **p < .01, ***p < .001). The § symbol designates the statistical tests were conducted on Box Cox transformed data. See also **Figures S7 and Table S2.**

In spleens, we found comparable relative numbers of CD4^+^ T-cells, but a significant effect of treatment on CD8^+^ T-cells with lower relative numbers in AOM/DSS-treated mice (Figure 4a). We found significant effects of treatment, environment and their interaction on the relative number of memory (CD44^+^) type within CD4^+^ T-cells in spleens. Pairwise comparisons showed significantly higher relative numbers of memory-type CD4+ T-cells in FerE- and FerL- than in Lab- mice. The differences in treatment were largely driven by higher relative numbers in the AOM/DSS-treated FerL+ and Lab+ than FerL- and Lab- mice, respectively (Figure 4a). Moreover, we found that the effect of environments on relative numbers of memory (CD44^+^) phenotype in CD8^+^ T-cells were driven by differences between the AOM/DSS-treated groups, where FerE+ and FerL+ showed higher numbers than Lab+ (Figure 4a).

In mLNs, we detected significant effects of treatment on both CD4^+^ and CD8^+^ T-cells, of which the CD4^+^ T-cells were higher and CD8^+^ T-cells lower in the AOM/DSS-treated mice (Figure 4c). The memory-type of CD4^+^ T-cells in mLNs were higher in FerL- than in FerE- and Lab- (Figure 4c). We also found an effect of environment on memory-type CD8^+^ T-cells in the mLNs of Fer mice compared to Lab mice (Figure 4c). Likewise, KLRG1 expressing T-cells (indicating antigenic experience) were increased in the Fer groups (Figure 4c).

Regulatory T-cells (Tregs) are important in conveying immunological tolerance to gut commensals.^44^ Yet, in malignancies like cancer, Tregs have been shown to interfere with a proper anti-tumor immune response.^45^ We observed no significant differences in relative numbers of splenic Tregs across environment or treatment (Figure 4b). In mLNs, we detected a significant interaction effect of environment and treatment on relative numbers of Tregs in mLNs driven by an increase in Lab+ mice compared to FerL+ mice (Figure 4d).

In the spleen, relative numbers of NK cells were similar across groups (Figure 4e). In mLNs, we found a significant effect of environment and treatment on relative number of NK cells, with pairwise comparisons showing significantly higher relative numbers in control-treated mice compared to AOM/DSS-treated mice, but no significant differences across environments (figure 4f). We detected significant interaction effects on KLRG1^+^ NK cells in both the spleens and mLNs, largely driven by higher numbers in FerE+ and FerL+ than in Lab+ mice (Figure 4(e,f)). Murine NK cells can be divided into maturation stages based on expression of CD11b and CD27, where the early (S1), mid (S2), late (S3) and fully mature (S4) stages corresponds to CD27^−^CD11b^−^, CD27^+^CD11b^−^, CD27^+^CD11b^+^ and CD27^−^CD11b^+^, respectively.^28,46^ In mLNs, but not in spleens, we found a significant effect of environment on the S4/S2 ratio (the two dominating subsets), with pairwise comparisons showing a significant higher ratio in FerE+ and FerL+ mice compared to Lab mice (Figure 4(e,f)).

We addressed whether feralization influenced T-cells and NK cells potencies as effector cells by assessing the production of IFNγ. Cells isolated from mLNs and incubated with PMA and Ionomycin, or IL-2 and IL-12, followed by flow cytometric evaluation of T- and NK cells with respect to IFNγ expression. We found a significant effect of environment on the frequency of IFNγ positive CD8^+^ T-cells, which were higher in FerE and FerL mice compared to Lab mice (Figure 4g). Moreover, we found a significant effect of treatment on IFNγ^+^ CD4^+^ T-cells, with higher relative numbers in AOM/DSS-treated mice than in control-treated mice. No significant effects of environment nor treatment were found for IFNγ^+^ NK cells (Figure 4g).

Taken together, the immunophenotyping data suggests that feralization in a farmyard-type environment promote immune maturation of the T and NK cell populations both locally (mLNs) and systemically (spleen).

## Discussion

Free-living mammals, including humans and mice, are exposed to a diverse range of microbes over their lifetime, which their immune system relies upon for development. Yet, disease modeling in mice usually take place under strictly hygienic conditions, far away from the typical lifestyle of the end goal for such studies, humans. To close the gap between the preclinical mouse model and human lifestyles, we have established a system where laboratory mice are raised under a full set of environmental conditions present in a naturalistic, farmyard-like habitat in indoor facilities.^34^ In the current study, we addressed the effects of housing lab mice in a farmyard-type habitat on development of colorectal cancer (CRC). We demonstrate that feralization in this environment had prominent clinical consequences in conferring protection toward colorectal carcinogenesis in the genetic (A/J Min/+ mice) as well as the chemical induction (AOM/DSS) models of CRC.

Our findings corroborate previous reports showing direct links between modulations of gut microbiotas and reduced colorectal carcinogenesis in AOM/DSS-treated mice^29,47–49^ and Min/+ mice,^50,51^ and indicate that the beneficial colorectal cancer-protective effects of the diverse farmyard-type habitat may be driven by the gut microbiota. We show that feralization led to shifts in gut microbiota profiles in both A/J and B6 mice, albeit differently in the two trials. Nevertheless, our study is not unique with respect to discrepancies in gut microbiota composition in naturalized mice, and this likely reflects differential sources for the natural microbes. Our A/J Min/+ mice were feralized in an environment containing farm material identical to those in our previous report of feral and feralized co-housed mice,^34^ and the Proteobacteria enrichment and increased *alpha*-diversity in all feralized A/J mice corresponded to our findings in both feral and feralized B6 mice in that report. Moreover, the findings from the feralized A/J mice corresponded well with previous reports from lab mice housed or engrafted with material from free-living mice,^29,34^ pet-store mice,^32^ and lab mice exposed to natural soil.^52^ In contrast, the B6 mice subjected to AOM/DSS treatment were feralized in an environment with components from a different farm source. In these feralized B6 mice, no changes in the relative abundance of Proteobacteria or in species richness was detected, corresponding to findings of re-wilded mice in outdoor facilities.^33^ The higher relative abundance of Bacteroidetes detected in the feralized B6 mice also complements previous findings in re-wilded mice,^33^ as well as lab mice engrafted with material from free-living mice,^29^ yet contrasts with our previous report of feral and feralized mice.^34^

Gut microbes associated with CRC vary greatly between studies and experimental setups. Although a lower relative abundance of Firmicutes was observed in our feralized B6 mice, OTUs with closest sequence similarities to *Lactobacillus* and *Limosilactobacillus* species were enriched. *Lactobacillus* has been reported to be predictive of a light tumor burden in the AOM/DSS model and various *Lactobacillus* strains have been shown to reduce gastrointestinal inflammation.^48^ However, *Bifidobacterium* strains have also been shown to confer anticancer effects,^53^ and we found higher relative abundance of this genus in lab B6 mice. Moreover, *Lachnospiraceae*, Clostridiales, Proteobacteria, *Alistipes* and *Aneroplasma* are all examples of taxa for which high baseline relative abundance has been associated with increased tumor burden in AOM/DSS model.^48^ In our AOM/DSS experiment, several OTUs with highest similarity to *Lachnospiraceae* spp., such as *Lacrimispora spp., Ruminococcus gnavus, Cuneatibacter caecimuris*, and *Stomatobaculum longum*, were enriched in lab B6 mice. Yet, *Alistipes* spp. and Proteobacteria were enriched in feralized B6 mice. These findings were not consistent with observations from the A/J Min/+ trial, emphasizing that different community structures could confer beneficial effects in different models of carcinogenesis.

Microbiota-associated dysregulation of immune pathways and the epithelial barrier are known drivers of carcinogenesis.^17^ Thus, a modulation of responses to inflammatory stimuli as previously implied in similar studies of a naturalized mouse microbiota^29^ is a feasible rationale for protection seen in the feralized mice. However, assessment of the pathogenic pathways was beyond the scope of this study, and the inhibitory mechanisms associated with feralization require further investigation.

Interestingly, in comparison to lab B6 mice, the feralized B6 mice treated with AOM and DSS demonstrated a robustness of their gut microbiota. Generally, large microbial shifts are observed in mice subjected to AOM and/or DSS treatment,^37,38,49^ and tumor burden has been associated with the magnitude of changes in gut microbiota community structure.^54^ We found major changes in the microbiota profile of lab mice, but only minor in feralized mice, following AOM/DSS treatment. A recent study by Rosshart *et al*. showed that a wild mouse microbiota was stable and resilient against external disturbances.^30^ Given the complex and diverse nature of gut microbes, it is feasible that the overall resilience of the gut microbiota, rather than single populations, is beneficial in preventing unhealthy states.^55^ Accordingly, it is possible that the feralized B6 microbiota is resilient to the perturbations inflicted by AOM and DSS, which may have contributed to the protective effects.

In the AOM/DSS trial, we assessed a panel of fecal SCFAs. As products of bacterial fermentation in the gut, SCFAs are known to play important roles in colonic energy metabolism, immune system, and gut barrier function. SCFAs, particularly butyrate, have been highly associated with colon health and anti-tumor properties,^17,56^ albeit with disagreement between studies.^57^ Previous studies have shown beneficial effects of SCFA administration on AOM- and DSS-induced carcinogenesis,^58^ and exacerbated carcinogenesis in SCFA-receptor deficient mice.^59^ However, we did not observe that feralization increased SCFA excretion, nor that protective mechanisms in our experiment were dependent of SCFAs. Nevertheless, analysis of the gut microbiota over-time indicated that known butyrate producers were reduced following AOM/DSS treatment in the lab mice. This suggests that SCFA levels may have been reduced which may have contributed to the exaggerated CRC development.

Immunophenotyping of T and NK cells showed increased expression of maturation markers, such as CD44 and KLRG1, in our feralized mice. The higher level of KLRG1^+^ NK cells found in feralized mice is similar to our previous findings in feralized co-housed mice.^34^ IFNγ-mediated responses are important in anti-tumor immunity and have been positively associated with survival in CRC,^60^ and the increased IFNγ response to stimuli in CD8^+^ T-cells in the feralized mice is also similar to our previous findings in feralized co-housed and feral mice.^34^ While these findings add consistency to the observed impact by feralization on immunity, more elaborate studies are needed to conclude about causal relationships with CRC protection.

Currently, the presented feralization model is unique in its ability to continuously expose mice to diverse environmental components, while allowing for controlled conditions such as light, temperature, and humidity. Moreover, this model enables control of the timing of encounter of various environmental stimuli, among them microbes, that could be valuable for future investigating the dynamics of host–microbe interactions. We emphasized this concept by including the late feralized mice in the AOM/DSS trial to investigate the potential role of feralization timing. Our late-feralized mice showed a disease phenotype in between the early-feralized and laboratory mice, yet closer to the former. Moreover, the gut microbiota composition was similar independently of feralization timing. These observations suggest that the transfer of maternal microbiota and early exposure to the farmyard environment had some effect yet was not essential in conferring protection against CRC.

While our study shows aspects of an original feralization approach, we do note some limitations. First, the two experiments reported here took place at two different sites with differences in the source of environmental material, mouse strain, age, gender, genotype, and use of different protocols in 16s rRNA sequencing. Hence, direct comparisons between the two trials presented within this manuscript should be made with caution. Nevertheless, it is noteworthy that similar disease outcomes were detected in both experiments. Second, the feralized mice described within this study are housed under a full set of complex environmental conditions. In the herein presented experiments, we have not assessed microbial components beyond the bacterial portion of the gut microbiota and certain pathogens and parasites, hence future studies should aim to unravel potential contributions of other communities such as fungi, viruses and bacteriophages. Moreover, the presence of a farmyard-type environment could offer other effects beyond modulations of the mouse microbiome. By testing the environmental material in both pens and in cages, we document limited effect of physiological and behavioral consequences of the enlarged space. Yet, nutritional elements, odors, tastes and other factors introduced through the farmyard-type environment remain uninvestigated. With studies of feralized mice, we are bringing the lab mice closer to a “real world” that has the potential to improve translational value to other mammals, including humans who rarely live in ultra-clean environments. The use of naturalized mouse studies is not intended to replace traditional reductionist studies, but rather complement them in search of both accuracy in reflecting true responses and precision in determining biological mechanisms.

In conclusion, we show that feralization of lab mice in a farmyard-type setting alleviate CRC development, and has considerable implications on gut microbiota and immunophenotype. We suggest that feralization of lab mice could complement traditional mice studies to improve our understanding of mechanisms underlying beneficial effects of diverse environments. The flexibility of choosing which factors to introduce, as well as the timing of their introduction, in the feralization model also provides novel opportunities to study dynamics of host interactions in various diverse environments.

## Materials and methods

### Animals and environmental settings

**Figure 5.**
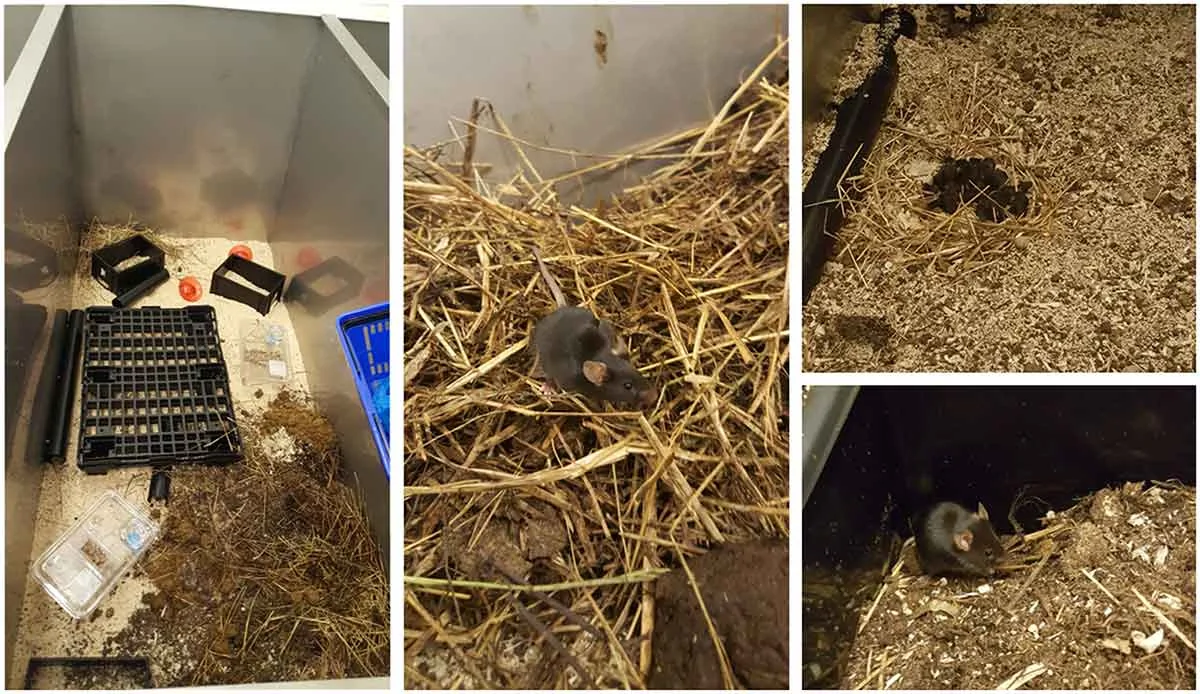
Photographs of the mouse pens and feralized B6 mice. The photographs show the layout of the mouse pens (left) and feralized B6 mice (right). The upper right photograph shows a nest of second generation feralized B6 mice

A microbially enriched, semi-naturalistic model was designed at the Norwegian University of Life Sciences. To resemble the common habitat of the house mouse (*Mus musculus*), indoor mouse pens containing natural environmental material were constructed. In the A/J Min/+ trial, pig pens (2.00 × 2.50 × 1.25 m) were adopted to house mice, containing sawdust, soil, compost, twigs, hay and fecal contents from pigs, cows and horses, as described previously.^34^ For the AOM/DSS trial, refinements to the model were made, and the feralization took place in specially designed mouse pens constructed of galvanized steel plates (1.10 × 2.40 × 1.20 m) with mouse igloos, running wheels, as well as plastic boxes and tunnels allowing for sheltering and nesting (Figure 5**; Video S1**). A base layer of woodchip bedding was laid down and enriched with organic soil (Plantasjen, Norway), straw, and fecal content from farmed pigs, cows, horses, and poultry, originating from an organic farm located in Eastern Norway. Initially, about 50 liters of fecal material, 40 liters of soil, and 80 liters of bedding was added to each of the four mouse pens. Every two weeks during the experiments, fresh farm animal fecal content (approximately 50 liters/pen), always from the same farm within each experiment, was added to the pens to simulate a natural situation and sustain the microbial load. Simultaneously with the addition, a portion of the old material was removed. The environmental material was kept moist with fresh tap water.

The mice were housed in either mouse pens (max 10/pen) or in individually ventilated cages (IVCs; Inovive Inc., San Diego, CA) (max 5/cage) with sterile bedding, mouse igloos and running wheels under standard conditions (12 h light/dark cycle, 23–25°C, 45–50% relative humidity). The non-feralized lab mice were kept under pathogen-free conditions. Water and standard chow diet (RM1(E), SDS; Special Diet Services, Witham, United Kingdom) were provided *ad libitum*. Throughout the trials, animal welfare was assessed by a health monitoring score sheet recording the animals’ bodyweight, rectal prolapse, rectal bleeding, general appearance and behavior daily. Animals exhibiting any symptom was kept under close observation. Humane endpoints were defined as follows: body weight loss >15%, rectal bleeding defined as blood around anus sustained over two subsequent days, a complete bulging of distal colon out of rectum, and severely under-conditioned appearance and behavior.

Animal experiments were approved by the Norwegian Animal Research Authority (FOTS IDs 6799 and 18012).

### A/J Min/+ model

A/J Min/+ (Min; multiple intestinal neoplasia) mice harbor a mutant allele of the murine *Apc* gene (adenomatous polyposis coli) and are thus predisposed to intestinal adenoma formation. On an A/J background, Min/+ mice consistently develop colonic adenomas, and are thus considered a relevant model of colorectal cancer in humans.^19,61,62^ The A/J Min/+ were B6 Min/+ mice (The Jackson Laboratories) back-crossed with wild-type A/J mice (The Jackson Laboratories). Breeding of A/J Min/+ mice at the Department of Experimental Biomedicine at NMBU, campus Adamstuen, has previously been described.^61^ Twenty male A/J Min/+ and twenty A/J wild-type (WT) aged 6–8 weeks were distributed to four age-matched groups (Figure 1a). The animals were either feralized in mouse pens or housed in conventional cages in a lab setting for 7–9 weeks before they were euthanized. One LabMin and one FerMin had bloody feces and altered behavior, and a tumor on the back, respectively, and were therefore euthanized earlier than the trial end. These two mice were excluded from all analyses, leaving n = 9 in the LabMin and FerMin groups. The age of the remaining mice at euthanasia ranged between 18 and 23 weeks. Animals were randomized to four days of harvesting, where all groups were represented each day. Tissues were collected after cervical dislocation.

### AOM/DSS model

Thirty female C57BL/6 JRj (B6; Janvier Labs, Saint-Berthevin Cedex, France) mice aged 3 weeks were acclimatized for one week under conventional, pathogen-free conditions in individually ventilated cages (IVCs) before being distributed to the different environments. The animals were feralized in mouse pens or housed in conventional cages in a lab setting for five weeks prior to breeding. The feralized females were mated with B6 males purchased from the same batch 2:1 in IVCs enriched with the same material as pens. After a 10-day breeding period, the females returned to the mouse pens to deliver. Additionally, 24 female mice from the same batch were housed and mated under pathogen-free conditions in IVCs.

Twenty-five female feralized offspring, and 45 female lab offspring, were included in the AOM/DSS trial. At 3 weeks of age, the offspring were weaned and randomly assigned to experimental groups (Figure 2a). At 7 weeks of age, colonic carcinogenesis was induced in the animals by use of a previously established protocol combining Azoxymethane (AOM; Sigma-Aldrich; 10 mg/kg) and repeated DSS (MP Biomedicals; 1% w/v, dissolved in distilled H_2_O) administration.^63^ Under transient gas anesthesia (isofluorane 3–4%, 200 mL/min), mice were either injected with AOM or sterile NaCl (B.Braun; 0.9%) subcutaneously into the neck skin fold. DSS (36,000–50,000 M.Wt.) was dissolved in distilled water prior to supply. A 1% DSS solution was supplied in three 7-day cycles (day 8–14, 31–38, 52–59), with a 16-day recovery period between the cycles. Fresh DSS solution was prepared and supplied every second day throughout the 7-day cycles. Control treatment entailed the same regimen with fresh distilled water only.

Due to late removal of a male pup from one of the mouse pens, four mice were potentially impregnated prior to the AOM/DSS treatment. These mice (one individual in FerE+, two in FerE- and one in FerL+ groups) were quarantined in cages enriched with the same material as the mouse pens for 12 days (day 12–24), while provided the same treatment as their respective groups. Bodyweight registrations from these mice a week before and during the quarantine were excluded from analyses, but data from these mice were included in the other analyses as we did not observe any signs of influence on outputs. One Lab mouse was found dead at week 16 and excluded from all analyses, leaving 14 mice in this group. The animals were sacrificed between 25 and 40 days after the last cycle of DSS/water administration. Animals were randomized to five days of harvesting, and all groups were represented each harvest day. Blood was collected by cardiac puncture while the animals were under terminal anesthesia induced by a single intraperitoneal (i.p.) injection of a cocktail consisting of Zoletil Forte (Virbac, Carros, France), Rompun (Bayer, Oslo, Norway), and Fentadon (Eurovet Animal Health, Bladel, The Netherlands) (0.1 mL/10 g body weight) with the following active ingredients: Zolezepam (32 mg/kg), Tiletamin (32 mg/kg), Xylazine (4.5 mg/kg) and Fentanyl 26 µg/kg). Tissues were collected after cervical dislocation.

### Pathogen screening and parasitology

For pathogen screening, blood was collected from three Lab mothers and six feralized mothers from the mouse pens by cardiac puncture while the animals were under terminal anesthesia induced by a single i.p. injection of a ZRF cocktail as described above. Serum was isolated by leaving blood samples clot at room temperature for 1–2 hours, followed by centrifugation at 1000–3000xg for 5–10 minutes. 100 µL serum from each animal was screened for common pathogens by BioDoc (Hannover, Germany).

For parasitology assessment, fecal pellets from a total of 12 female offspring housed in clean cages (3 animals from each of 4 cages), 6 animals housed in cages enriched with the natural environmental material also found in mouse pens (3 animals from each of 2 cages) and 24 animals housed in mouse pens (6 animals from each of 4 pens) was collected. Pellets were pooled, resulting in one sample per cage and two samples per pens. Feces were examined for parasites by standard methods including McMasters counting technique, and immunofluorescence antibody test (IFAT), for *Giardia spp*. and *Cryptosporidium spp*.

### Scoring of intestinal lesions by surface microscopy

The colons were prepared as described previously.^61^ Briefly, each colon was fixated flat between two filter papers in formalin solution (VWR Chemicals; 10%, neutral buffered) for 24 hours prior to staining with Methylene blue solution (MB; Sigma-Aldrich; 0.1% in 10% formalin, neutral buffered). The colons were stored refrigerated in 70% ethanol until analysis. The identification of intestinal lesions was performed by microscopy according to previously described procedure.^19^ In short, an inverted light microscope (CKX41, Olympus Inc., Hamburg, Germany) equipped with a digital color camera (DP25, Olympus) was used to examine the colons for lesions. Aberrant crypt foci (ACF) stain bright blue/green and have enlarged crypts with compressed luminal openings, while normal crypts stain more subdued green/brown (**Figure S8A**). Thus, ACFs can be recognized and distinguished from normal epithelia. Diameters were measured using an eye piece graticule, and colonic lesion size (mm^2^) was calculated based on the measured diameters. The total number of lesions, lesion load and distribution were measured and calculated per mouse in order to study lesion development in the intestines. Lesion load (mm^2^) was defined as the sum of the area of all lesions observed in an intestine.

### Histopathological classification of intestinal lesions

Because scoring of ACFs by MB-staining is less characterized in B6 mice than A/J Min/+ mice, we subsequently conducted histopathological classification of lesions in colons from the AOM/DSS-treated FerE, FerL, and Lab groups. The six colons in each group with the largest mean lesion size determined by surface microscopy were selected for further examination. Swiss rolls of these colons were prepared by rolling lengthwise from oral to rectal end, with the mucosa facing inwards. The swiss rolls were embedded in paraffin, and for each paraffin-embedded colon, sections (2–3 μm thick) were made at three different depths (top, middle, and bottom) to detect lesions over the width of the flattened intestine. For two individuals in the Lab+ group, only two sections were assessed. The sections were stained with hematoxylin and eosin (H&E) and examined blindly by a pathologist using high-resolution digitized slides scanned by a Philips UFS slide scanner. Lesions were classified as hyperplasia/dysplasia, adenomas (tumors restricted to the mucosa) or carcinomas (tumors with distinct infiltrative growth through the mucosa into the submucosa) (**Figure S8B**).

### Microbial community analysis by 16S rRNA gene amplicon sequencing

In the A/J Min/+ trial, fecal pellets were snap frozen in liquid nitrogen immediately after collection and stored at -80°C until DNA extraction. DNA extraction and library preparation of the V3-V4 regions of the 16S rRNA gene was conducted at NMBU according to a previously described procedure.^64^ High-throughput amplicon sequencing was conducted on a MiSeq platform (Illumina Inc.) using V3 sequencing chemistry in a paired-end mode.

In the AOM/DSS trial, fecal pellets were collected in sterile tubes pre-filled with Zirconia-Silicate beads (0.1–0.15 mm, Cole-Palmer) and Stool DNA Stabilizer buffer (STRATEC Molecular GmbH). Samples were snap frozen in liquid nitrogen immediately after collection and stored at −80°C until DNA extraction. DNA was extracted as previously described,^65^ including mechanical lysis by bead-beating. Amplicon libraries were prepared via a two-step PCR amplifying the V3-V4 regions, as described in detail previously.^66^ Amplicons were purified with the AMPure XP system (Beckmann) before sequencing. High-throughput amplicon sequencing was performed at the ZIEL Institute for Food & Health, Technical University of Munich, according to previously described procedures.^65^ Sequencing was carried out in a paired-end mode (PE300) using a MiSeq system (Illumina Inc.).

Raw reads were processed with the Integrated Microbial Next Generation Sequencing pipeline,^67^ based on the UPARSE approach.^68^ Briefly, sequences were demultiplexed, trimmed to the first base with a quality score >3, and assembled. Sequences with <300 and >600 nucleotides (AOM/DSS trial; paired-end analysis) or <200 and >300 nucleotides (A/J Min/+ trial; single-end analysis), as well as assembled sequences with expected error >3 were excluded from the analysis (USEARCH 8.1 (AOM/DSS trial) or 8.0 (A/J trial).^69^ Remaining reads were trimmed by 10 nucleotides at forward and reverse end to prevent analysis of regions with distorted base composition. The presence of chimeras was tested with UCHIME.^70^ Operational taxonomic units (OTUs) were clustered at 97% sequence similarity (USEARCH 8.1),^69^ and only those with a relative abundance >0.25% in at least one sample were kept.^71^ Taxonomies were assigned at 80% confidence level with the RDP classifier^72^ (version 2.11, training set 15). Sequences were aligned with MUSCLE,^73^ and tree generated with Fasttree.^74^ Specific OTUs were identified using EzBioCloud.^75^

Raw sequence files were deposited to the Sequence Read Archive and are available under the accession number PRJNA669440.

### Short-chain fatty acid analysis

Analysis of short-chain fatty acids in stool samples was conducted using a Trace 1310 gas chromatograph (Thermo Scientific) equipped with an auto sampler, a flame ionization detector (FID), a split injector, and a Stabilwax DA column (Restek; 30 m, 0.25 mm ID, 0.25 µm), according to previously described procedures.^76^ Briefly, thawed fecal samples were dissolved in water and homogenized in Fastprep (MP Biomedicals). Supernatant was collected and mixed 1:1 (vol/vol) with internal standard (solution of 0.4% formic acid and 2000 µM 2-methylvaleric acid). Samples were centrifuged, and supernatant was transferred into spin columns (VWR; 0.2 µm filter) and centrifuged again. Eluates were transferred into GC vials and analyzed in the GC-FID instrument. The software Chromeleon (v. 7.2) was used for instrument control, quantification and data analysis. SCFA quantification was calculated based on a standard curve made from two-fold dilutions of SCFA standards.

### Immunophenotyping

Mesenteric lymph nodes (mLNs) and whole spleens were harvested and kept in RPMI-1640 medium (Sigma-Aldrich) with 2% FCS on ice until extraction of cells. Cells were extracted from tissues using GentleMACS dissociator. For splenic tissue, a collagenase/DNAse solution was used for digestion. Splenic suspensions were briefly treated with NH_4_Cl solution to lyse erythrocytes. Single-cell suspensions were prepared by running through a 70 µm cell strainer (BD Biosciences) and concentrations standardized using Countess II automated cell counter (Thermo Fischer Scientific).

Immunophenotyping was carried out on ice by incubating single-cell suspensions in RPMI medium with 0.5% BSA. Following Fc blocking with anti-CD16/CD32 antibody, cells were stained with Fixable Live/Dead Yellow (Thermo Fisher) and incubated with combinations of monoclonal antibodies listed in **Table S2**. For intracytoplasmic staining, surface staining was followed by additional steps of treatment with Intracellular Fixation & Permeabilization buffer or Foxp3 Staining buffer (eBioscience) according to manufacturer’s manual. Cells were analyzed using a Gallios 3-laser flow cytometer and Kaluza 1.2 software (Beckman Coulter). Gating strategies are depicted in **Figure S7**.

### Ex vivo activation of immune cells

Cells isolated from mLNs were seeded on 96-well plates (500,000 cells/well) in triplicates. The cells were incubated with a cocktail of Brefeldin A (Sigma-Aldrich) in RPMI medium with PMA (phorbol 12-myristate-13-acetate; Sigma-Aldrich) and ionomycin for T-cell activation, and murine IL-2 and IL-12 for NK cell stimulation. Cells were incubated for 4 hours, spun down and stained for immunophenotyping of intracytoplasmic IFNγ as described above.

### Statistical analyses

Microbial profiles and composition were analyzed in the R programming environment (R version 4.0.2)^77^ using Rhea (available from: https://github.com/Lagkouvardos/Rhea).^78^ OTU tables were normalized to account for differences in sequence depth by division to their sample size and then multiplication by the size of the smaller sample. *Beta*-diversity was computed based on generalized UniFrac distances,^79^ and the significance of separation between groups was tested by permutational multivariate analysis of variance (PERMANOVA). *Alpha*-diversity was assessed based on species richness and Shannon effective diversity as explained in detail in Rhea. Only taxa with a prevalence of ≥30% (proportion of samples positive for the given taxa) in one given group, and relative abundance ≥0.25% were considered for statistical testing. Statistical differences in abundance and prevalence between two groups were determined by Wilcoxon Rank Sum test and Fisher’s Exact test, respectively. Statistical differences in abundance and prevalence between ≥3 groups were determined by Kruskal-Wallis followed by Wilcoxon Rank Sum tests, and Fisher’s Exact tests, respectively. *P*-values were corrected for multiple comparisons by the Benjamini–Hochberg method. Analyses of over-time differences in abundance and prevalence (within groups) was assessed by Wilcoxon Signed Rank Sum test and Fisher’s test, respectively.

Statistical analyses were performed using the R programming environment, JMP Pro 15 (v15.2.1; SAS Institute Inc.; Cary, NC, USA) or GraphPad Prism 6 (v6.07; GraphPad Software Inc.; San Diego, CA, USA). All applied statistical methods are specified in figure legends. Prior to application of parametric statistics, normality and homogeneity of variance was tested on residuals by Shapiro-Wilk and Levene’s tests, respectively. Heatmaps were generated using the *heatmap.2* function from the *gplots* package^80^ in R. Figures were created using GraphPad Prism 6 and Inkscape (v0.92.4; http://www.inkscape.org/).

## Supplementary Material

Supplemental Material

## Data deposition

Raw sequence files can be found in NCBI’s Sequence read archive (SRA) under the accession number PRJNA669440.
