# Supplementary material for: Naturalizing laboratory mice by housing in a farmyard-type habitat confers protection against colorectal carcinogenesis
Source: Gut Microbes. 2021 Nov 9;13(1):1993581. doi: 10.1080/19490976.2021.1993581 (PMC8583187; doi:10.1080/19490976.2021.1993581)
Supplement: Supplemental Material [file KGMI_A_1993581_SM4446.zip › Supplementary Figures caption.docx]

# **Supplementary material**

## Figures S1-S8

**Figure S1**. Extended data from the A/J Min/+ trial. **(A)** Dot plot of bodyweight for Fer and Lab A/J Min/+ and WT mice at baseline (t0) and endpoint (t1). **(B)** Assessment of small intestinal lesions in Fer and Lab A/J Min/+ mice at endpoint. The occurrence of lesions is presented as total number and mean size. Box plots show median (line), mean (+), IQR (box) and minimum to maximum (whiskers). **(C)** Heatmap of relative abundance of specific OTUs enriched in FerWT and LabWT mice at endpoint (t1). OTUs of which abundance or prevalence differed significantly between the groups (determined by Wilcoxon Rank Sum and Fisher’s exact tests, respectively) are plotted. Blue color indicates the OTUs were absent or below cutoffs for analyses. Blue color indicates the OTUs were absent or below cutoffs for analyses. The bacterial species with a valid name closest to the corresponding OTUs is indicated along with its sequence similarity; those OTUs identifiable at the species level (≥97 % similarity) are written in bold letters. Phyla to which the OTUs belong are designated with colored squares as specified in Figure 1 E. Frames indicate significant increased abundance or prevalence in FerWT (brown) and LabWT (grey).

**Figure S2**. Rarefaction curves. The curves show number of observed species in each sample plotted against the number of reads for analyzed fecal samples from the **(A)** AJ/ Min/+ and **(B)** AOM/DSS trials. The curves were generated by normalized counts via simple division to their sample size followed by multiplication by the size of the smaller sample, using Rhea^79^. The five top undersequenced samples are marked in red, with group and timepoint indicated.

**Figure S3**. Extended data from the AOM/DSS trial. **(A)** Bodyweight curves for control (NaCl/H_2_O treated) animals, presented as per cent of initial body weight. Box plots show median (line), mean (+), IQR (box) and minimum to maximum (whiskers). Significant changes in bodyweight from first to last day of the trial (0 to 80), and first to last day of each cycle (8-14, 31-38, 51-59), were determined using repeated measures ANOVA with Tukey multiple comparison tests and indicated in the figure. **(B)** Colonic lesion assessment of control (NaCl/H_2_O treated) animals, presented as number of lesions, mean size of lesions and lesion load. (**C)** Scheme of the experimental setup showing timeline and grouping of Fer mice confined to cages. At trial week 7, a small sub-population of FerE and FerL animals (Figure 2A) were moved to cages enriched with the same environmental material as the mouse pens, constituting the two confined space (FerE^cage^ and FerL^cage^) groups. Samples were collected at baseline (t0; week 0) and endpoint (t1; week 7-9). ‡ one individual deceased before endpoint and were consequently excluded from lesion scoring. **(D)** Bodyweight curves for AOM/DSS treated FerE mice confined to cages FerE^cage^ compared to the corresponding pen-housed group (FerE+). Data presentation and statistics as in A. **(E)** Bodyweight curves for AOM/DSS treated FerL mice confined to cages FerL^cage^ compared to the corresponding pen-housed group (FerL+). Data presentation and statistics as in A. **(F)** Scoring of colonic lesions in the FerE^cage^ and FerL^cage^ groups, presented as number of lesions, mean size of lesions and lesion load, compared to the corresponding pen-housed groups FerE+ and FerL+. Statistical differences were determined by unpaired t-tests. **(G)** Fluid intake during DSS or H_2_O administration. Bars represent estimated mean intake per animal, calculated by drinking bottle volumes for each housing unit, presented as mean intake per animal for the whole treatment regimen with the standard deviation (SD) shown via the whiskers. Black bars show groups administered DSS. White bars show groups administered H_2_O water. Lab+ include 3 cages, n=15; FerE/L+ include 3 mouse pens, total n=29; FerE/L- include 1 pen, total n=10; FerE/L^cage^+ includes 2 cages, total n=9.

**Figure S4**. Pathogen screening. Serum-No. 1752, 1761, 1762, 1771, 1772 and 1781 represent Fer mice. Serum-No. 1784, 1795 and 1805 represent Lab mice.

**Figure S5**. Pairwise PERMANOVA comparisons of microbiota profiles for AOM/DSS treated feralized and laboratory mice. Pairwise multi-dimensional scaling (MDS) plots of microbiota profiles and differences for calculated for: (**A**) Baseline (t0) between groups, **(B)** baseline to endpoint change within groups, and (**C**) endpoint (t1) between groups. Differences were calculated using generalized UniFrac distances in Rhea ^79^. *P*-values are corrected for multiple testing using the Benjamini-Hochberg method. (**D**) Heatmap of relative abundance of specific OTUs enriched in AOM/DSS treated Fer and Lab mice at endpoint (t1). The occurrence of OTUs for which the relative abundance or prevalence differed significantly between the groups (determined by Kruskal-Wallis and Fisher’s exact test, respectively) are plotted. Blue color indicates the OTUs were absent or below cutoffs for analyses. The bacterial species with a valid name closest to the corresponding OTUs is indicated along with its sequence similarity; those OTUs identifiable at the species level (≥97 % similarity) are written in bold letters. Phyla to which the OTUs belong are designated with colored squares as specified in Figure 1 C. Frames indicate significant increased relative abundance or prevalence in FerE (green), FerL (orange) and Lab (blue) compared to one of the other groups determined by pairwise analyses (Wilcoxon Signed Rank Sum/Fisher’s Exact tests with Benjamini-Hochberg correction for multiple comparisons).

**Figure S6.** Extended gut microbiota characterizations from the AOM/DSS trial. **(A)** Multi-dimensional scaling (MDS) plot of fecal microbiota profiles (generalized UniFrac distances) for control (NaCl/H_2_O)-treated and corresponding AOM/DSS-treated groups at baseline (t0) and endpoint (t1). Significance of separation was determined by PERMANOVA. d=distance scale. (**B**) Observed number of OTUs (Richness) and Shannon Effective diversity index for control-treated groups. Box plots show median (line), meanmean (+), IQR (box) and minimum to maximum (whiskers). (**C**) Taxonomic binning at the rank of phylum, presented as relative abundance for each individual in the control-treated groups. (**D**) Multi-dimensional scaling (MDS) plot of fecal microbiota profiles (generalized UniFrac distances) for cage-housed Fer mice (FerE^cage^ and FerL^cage^; Figure S3C) and corresponding AOM/DSS-treated groups at baseline (t0) and endpoint (t1). (**E**) Observed number of OTUs (Richness) and Shannon Effective diversity index for cage-housed Fer mice. Box plots show median (line), mean (+), IQR (box) and minimum to maximum (whiskers). (**F**) Taxonomic binning at the rank of phylum, presented as relative abundance for each individual in the cage-housed Fer mice.

**Figure S7**. Immunophenotyping gating strategies. (**A**) Single cell, mononuclear cell (MNC) and live cell gating. (**B**) NK cells were defined as NK1.1^+^ CD3^-^ cells and T-cells as NK1.1.^-^ CD3^+^. Subsets gated on NK cells and T-cells are indicated by arrows. For NK cells were gated for KLRG1 expression, and combinations of CD27 and CD11b expression. (**C**) Regulatory T-cells (Tregs) were gated on CD4^+^ T-cells (defined as CD4^+^ CD3^-^ % of Live CD45^+^) and defined as CD25^+^ FOXp3^+^ % of Live CD45^+^. Tregs were further gated for expression of KLRG1.

**Figure S8.** Identification of colonic lesions in (**A**) MB-stained colons and (**B**) H&E-stained Swiss roll sections.
