# Supplementary material for: Naturalizing laboratory mice by housing in a farmyard-type habitat confers protection against colorectal carcinogenesis
Source: Gut Microbes. 2021 Nov 9;13(1):1993581. doi: 10.1080/19490976.2021.1993581 (PMC8583187; doi:10.1080/19490976.2021.1993581)
Supplement: Supplemental Material [file KGMI_A_1993581_SM4446.zip › supplementary tables.docx]

## Tables S1-S2

**Table S1**. Data presented in Figure 3E; Enriched and reduced OTUs among the three groups in response to AOM/DSS treatment (over-time analysis within groups).

|  | **Closest species name (% similarity)** | **Abundance** | | | **Prevalence** | | | **Paired Wilxocon, *P*=** | | | **Fisher's Exact, *P*=** | | |
| --- | --- | --- | --- | --- | --- | --- | --- | --- | --- | --- | --- | --- | --- |
|  |  | FerE+ | FerL+ | Lab+ | FerE+ | FerL+ | Lab+ | FerE+ | FerL+ | Lab+ | FerE+ | FerL+ | Lab+ |
| OTU_1 | *Alloprevotella rava* (91.6 %) |  |  | ↑ |  |  |  |  |  | 0.0269 |  |  |  |
| OTU_10 | *Paramuribaculum intestinale* (91.1 %) | ↑ |  | ↑ |  |  |  | 0.0245 |  | 0.0245 |  |  |  |
| OTU_11 | *Desulfovibrio vulgaris* (89.9 %) | ↑ |  | ↓ |  |  |  | 0.0302 |  | 0.0398 |  |  |  |
| OTU_113 | ***Faecalibaculum rodentium* (97.8 %)** |  |  |  |  |  | ↑ |  |  |  |  |  | 0.0019 |
| OTU_124 | *Enterocloster lavalensis* (94.1 %) |  |  |  |  |  | ↓ |  |  |  |  |  | 0.0063 |
| OTU_13 | ***Alistipes dispar* (97.5 %)** |  |  | ↓ | ↑ |  | ↓ |  |  | 0.0313 | 0.0028 |  | 0.0128 |
| OTU_150 | *Lacrimispora saccharolytica* (95.5 %) |  |  |  |  |  | ↓ |  |  |  |  |  | 0.0063 |
| OTU_1590 | *Hungatella xylanolytica* (94.5 %) |  |  |  |  |  | ↓ |  |  |  |  |  | 0.0329 |
| OTU_16 | *Muribaculum intestinale* (90.2 %) |  | ↑ | ↑ |  |  | ↑ |  | 0.0313 | 0.0078 |  |  | 0.0159 |
| OTU_17 | *Duncaniella dubosii* (92.9 %) |  |  |  |  |  | ↑ |  |  |  |  |  | 0.0013 |
| OTU_174 | *Muribaculum intestinale* (91.8 %) |  |  |  |  |  | ↑ |  |  |  |  |  | 0.0128 |
| OTU_18 | ***Akkermansia muciniphila* (99.8 %)** |  |  |  | ↑ | ↑ | ↑ |  |  |  | 0.0007 | 0.0001 | 0.0002 |
| OTU_19 | ***Muribaculum intestinale* (99.5 %)** | ↑ | ↑ | ↑ |  |  |  | 0.0413 | 0.0006 | 0.0006 |  |  |  |
| OTU_20 | *Anaeroplasma bactoclasticum* (91.8 %) |  |  |  | ↑ | ↑ |  |  |  |  | 0.0000 | 0.0000 |  |
| OTU_2297 | *Duncaniella dubosii* (90.4 %) |  |  |  |  |  | ↑ |  |  |  |  |  | 0.0000 |
| OTU_23 | *Muribaculum gordoncarteri* (91.4 %) |  |  | ↑ |  |  |  |  |  | 0.0005 |  |  |  |
| OTU_24 | *Dubosiella newyorkensis* (89.4 %) |  |  |  |  | ↑ | ↑ |  |  |  |  | 0.0329 | 0.0000 |
| OTU_25 | ***Ruminococcus gnavus* (97.6 %)** |  |  |  |  |  | ↑ |  |  |  |  |  | 0.0044 |
| OTU_26 | ***Limosilactobacillus reuteri* (99.8 %)** |  | ↓ |  |  |  |  |  | 0.0105 |  |  |  |  |
| OTU_3 | ***Alistipes timonensis* (98.0 %)** |  |  | ↑ |  |  |  |  |  | 0.0049 |  |  |  |
| OTU_33 | *Muribaculum gordoncarteri* (88.6 %) |  |  | ↑ |  |  |  |  |  | 0.0002 |  |  |  |
| OTU_36 | ***Anaerocolumna cellulosilytica* (97.1%)** |  |  |  |  | ↓ |  |  |  |  |  | 0.0183 |  |
| OTU_39 | *Paramuribaculum intestinale* (91.8 %) |  |  | ↑ |  |  |  |  |  | 0.0161 |  |  |  |
| OTU_4 | *Alistipes finegoldii* (94.8 %) | ↓ |  |  |  |  |  | 0.0043 |  |  |  |  |  |
| OTU_42 | *Stomatobaculum longum* (95.5 %) |  |  |  |  |  | ↓ |  |  |  |  |  | 0.0002 |
| OTU_43 | ***Phocaeicola sartorii* (99.8 %)** |  |  |  | ↑ |  |  |  |  |  | 0.0002 |  |  |
| OTU_45 | ***Flintibacter butyricus* (98.3 %)** |  |  | ↓ |  |  |  |  |  | 0.0059 |  |  |  |
| OTU_46 | ***Turicimonas muris* (99.3 %)** |  |  |  |  |  | ↑ |  |  |  |  |  | 0.0006 |
| OTU_476 | *Alistipes putredinis* (96.4 %) |  | ↓ |  |  |  |  |  | 0.0419 |  |  |  |  |
| OTU_48 | *Paramuribaculum intestinale* (91.1 %) | ↓ |  |  |  |  | ↑ | 0.0479 |  |  |  |  | 0.0004 |
| OTU_497 | *Lacrimispora aerotolerans* (95.9 %) |  |  |  |  |  | ↓ |  |  |  |  |  | 0.0001 |
| OTU_5 | ***Kineothrix alysoides* (97.4 %)** |  | ↓ | ↓ |  |  |  |  | 0.0420 | 0.0010 |  |  |  |
| OTU_51 | *Muribaculum intestinale* (91.8 %) | ↑ |  |  |  |  |  | 0.0029 |  |  |  |  |  |
| OTU_52 | *Flintibacter butyricus* (92.2 %) |  |  |  |  |  | ↓ |  |  |  |  |  | 0.0000 |
| OTU_55 | ***Ruthenibacterium lactatiformans* (97.1 %)** |  |  |  |  |  | ↓ |  |  |  |  |  | 0.0003 |
| OTU_56 | *Muribaculum gordoncarteri* (91.6 %) |  | ↓ |  |  |  |  |  | 0.0391 |  |  |  |  |
| OTU_57 | ***Bifidobacterium animalis subsp. Animalis* (99.8 %)** |  |  |  |  |  | ↑ |  |  |  |  |  | 0.0000 |
| OTU_59 | *Clostridium polysaccharolyticum* (93.8 %) |  |  |  |  |  | ↓ |  |  |  |  |  | 0.0000 |
| OTU_63 | ***Campylobacter jejuni subsp. Doylei* (100 %)** |  |  |  | ↓ |  |  |  |  |  | 0.0022 |  |  |
| OTU_703 | *Lacrimispora aerotolerans* (96.7 %) |  |  |  |  |  | ↓ |  |  |  |  |  | 0.0003 |
| OTU_9 | *Prevotella stercorea* (91.2 %) |  |  | ↑ |  |  |  |  |  | 0.0049 |  |  |  |
| OTU_90 | ***Kineothrix alysoides* (97.1 %)** |  |  |  |  |  | ↑ |  |  |  |  |  | 0.0058 |
| OTU_91 | ***Lawsonibacter asaccharolyticus* (98.3 %)** |  |  | ↓ |  |  | ↓ |  |  | 0.0156 |  |  | 0.0329 |
| OTU_97 | *Oscillibacter valericigenes* (94.8 %) |  |  |  |  |  | ↓ |  |  |  |  |  | 0.0003 |
| OTU_98 | *Eubacterium plexicaudatum* (96.9 %) |  |  |  |  |  | ↓ |  |  |  |  |  | 0.0407 |

**Table S2**. Details of antibodies used in immunophenotyping.

| **Antibody** | **Fluorochrome** | **Clone** | **Vendor** |
| --- | --- | --- | --- |
| CD11b | FITC | M1/70.15 | eBioscience/Affymetrix |
| CD27 | PE-Cy7 | LG.3A10 | BioLegend |
| CD3 | PCP-Cy5.5 | 17A2 | eBioscience/Affymetrix |
| CD4 | PE/Dazzle594 | GK1.5 | BioLegend |
| CD44 | FITC | IM7 | eBioscience/Affymetrix |
| CD45 | APC-e780 | 30-F11 | eBioscience/Affymetrix |
| CD62L | APC | MEL-14 | eBioscience/Affymetrix |
| CD8 | Alexa Fluor 700 | 53-6.7 | BD Biosciences |
| FOXp3 | PE | FJK-16s | eBioscience/Affymetrix |
| NK1.1 | PE | PK136 | BioLegend |
| NKp46 | e-660 | 29A1.4 | eBioscience/Affymetrix |
| Helios | FITC | 22F6 | BioLegend |
| KLRG1 | BV421 | 2F1 | BD Biosciences |
| IFNg | PE | XMG1.2 | eBioscience/Affymetrix |
