# Supplementary material for: Naturalizing laboratory mice by housing in a farmyard-type habitat confers protection against colorectal carcinogenesis
Source: Gut Microbes. 2021 Nov 9;13(1):1993581. doi: 10.1080/19490976.2021.1993581 (PMC8583187; doi:10.1080/19490976.2021.1993581)
Supplement: Supplemental Material [file KGMI_A_1993581_SM4446.zip › supplementary Video S1 caption.docx]

## Video S1

Video 1. Montage of short videos of feralized B6 mice in mouse pens.
