# Supplementary figures and images for: Naturalizing laboratory mice by housing in a farmyard-type habitat confers protection against colorectal carcinogenesis
Source: Gut Microbes. 2021 Nov 9;13(1):1993581. doi: 10.1080/19490976.2021.1993581 (PMC8583187; doi:10.1080/19490976.2021.1993581)

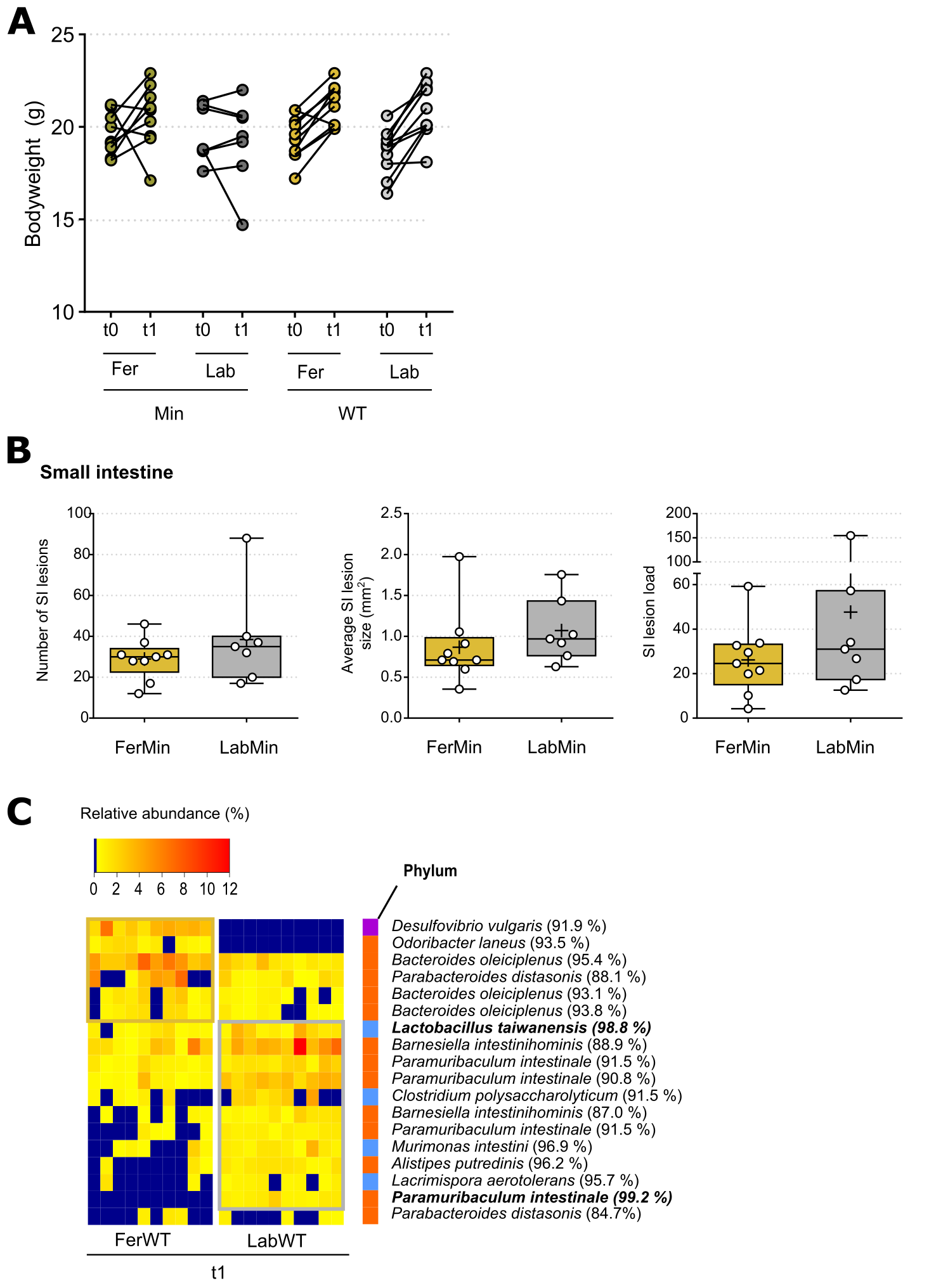

Supplement: Supplemental Material [file KGMI_A_1993581_SM4446.zip › SupplementaryFigureS1.tiff]

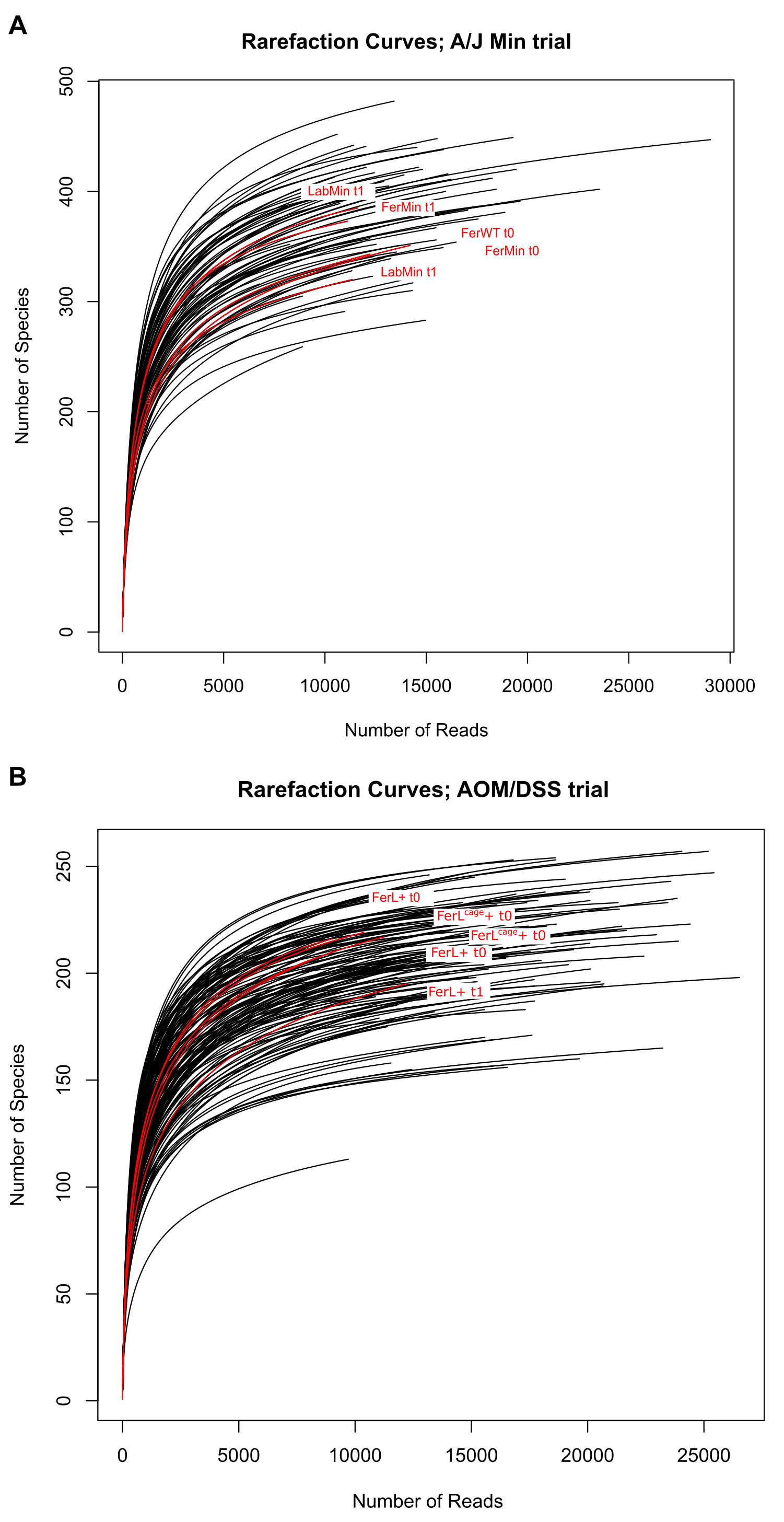

Supplement: Supplemental Material [file KGMI_A_1993581_SM4446.zip › SupplementaryFigureS2.tiff]

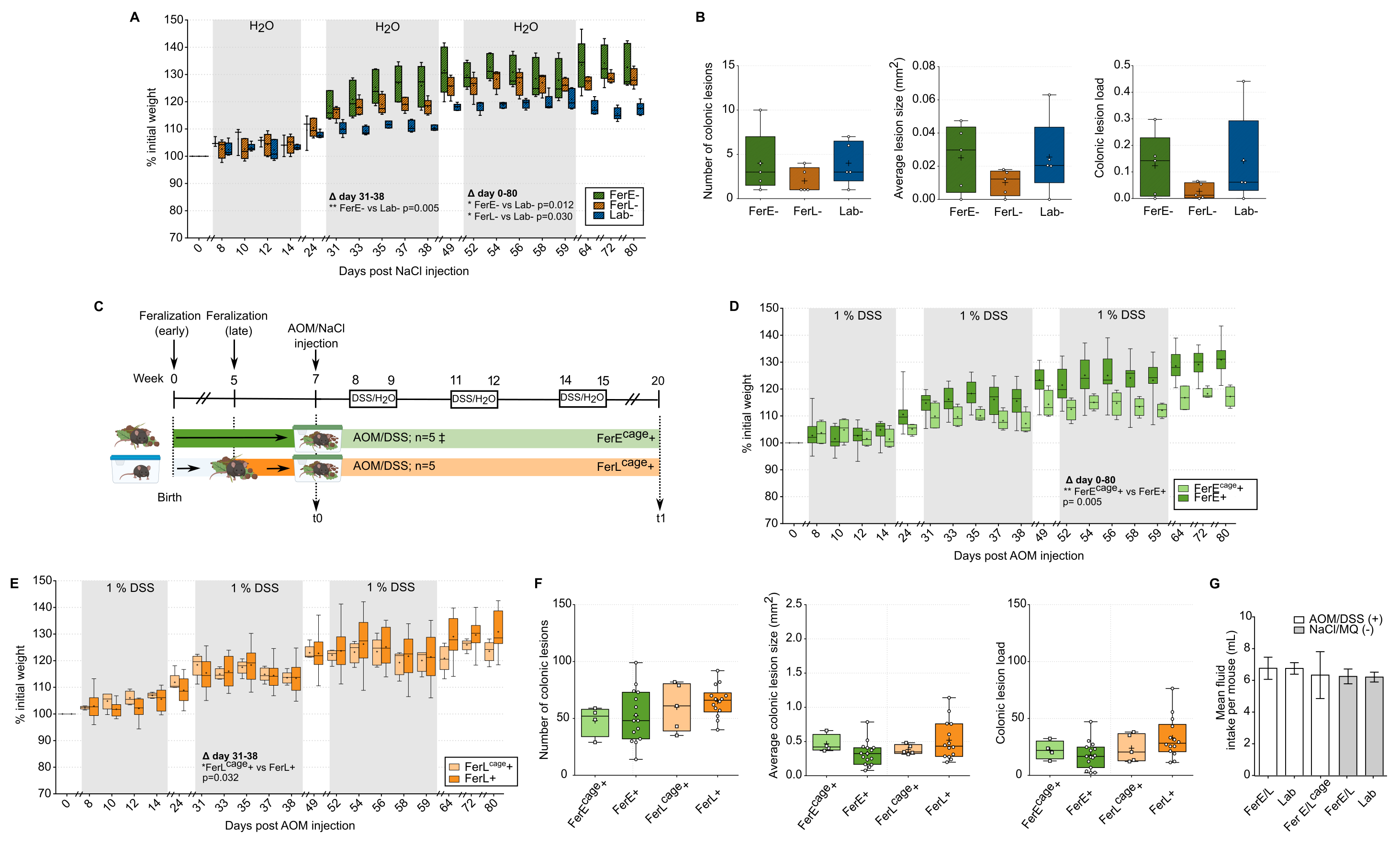

Supplement: Supplemental Material [file KGMI_A_1993581_SM4446.zip › SupplementaryFigureS3_rev1.tiff]

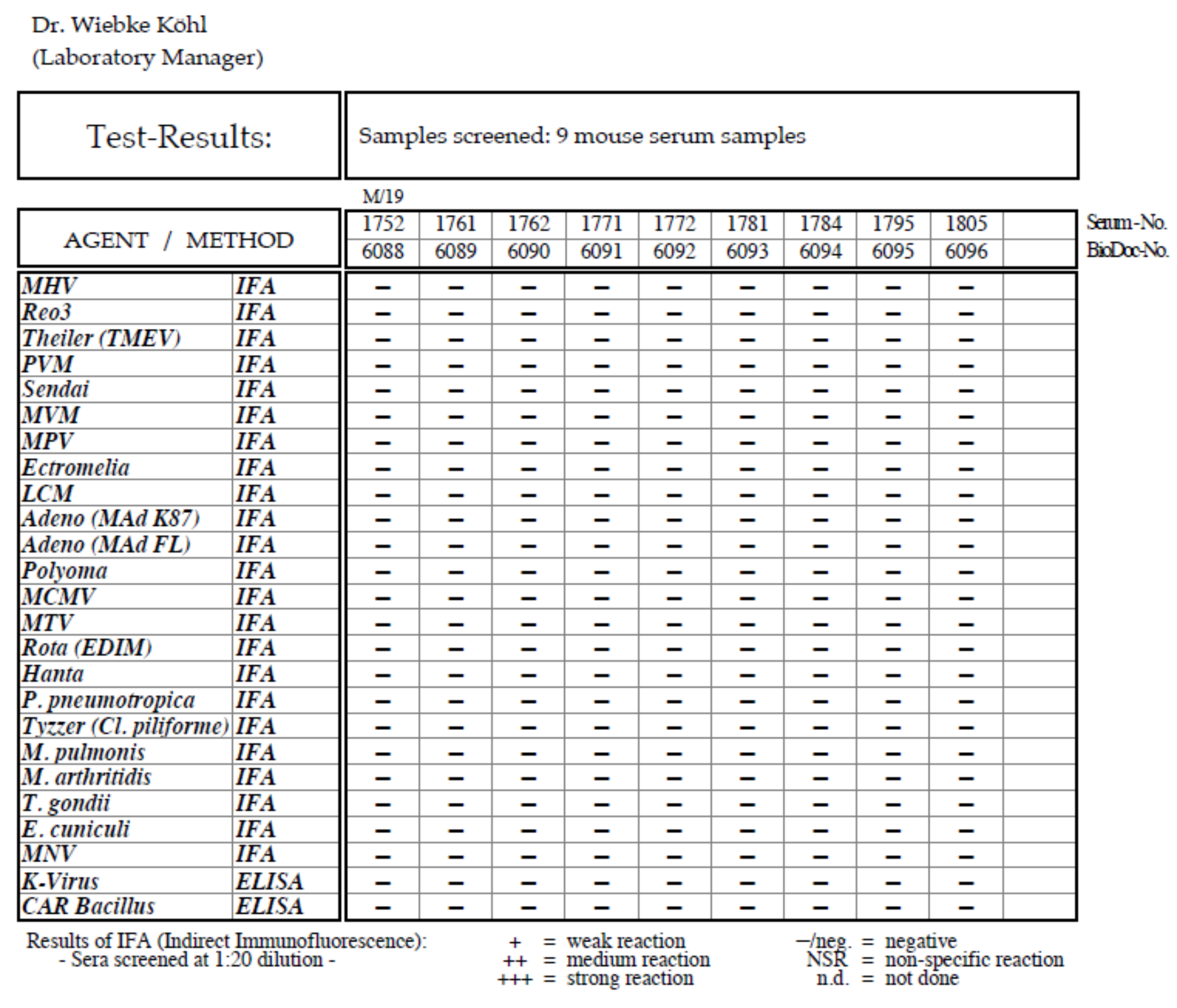

Supplement: Supplemental Material [file KGMI_A_1993581_SM4446.zip › SupplementaryFigureS4_rev1.tiff]

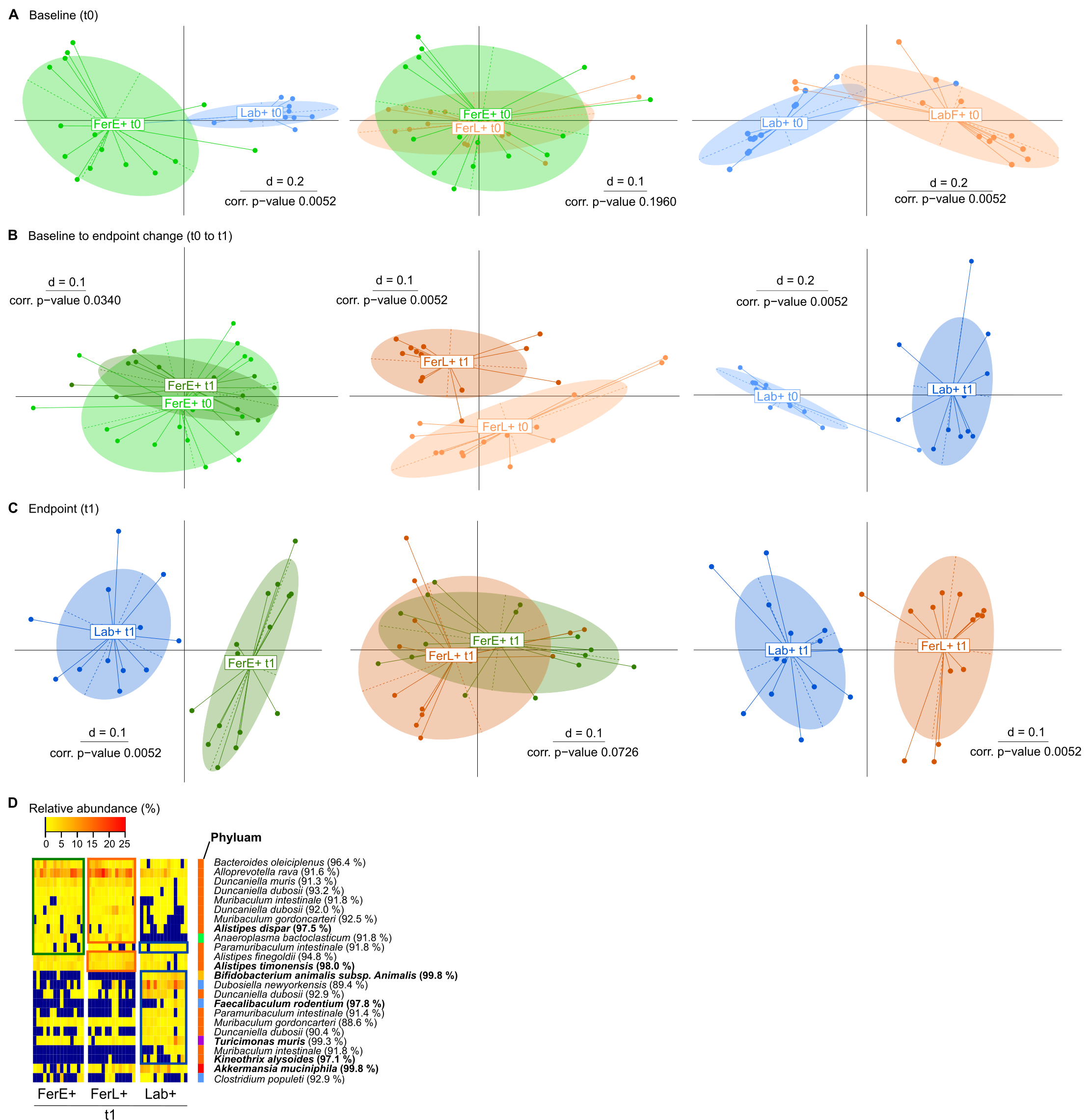

Supplement: Supplemental Material [file KGMI_A_1993581_SM4446.zip › SupplementaryFigureS5_rev1_2.tiff]

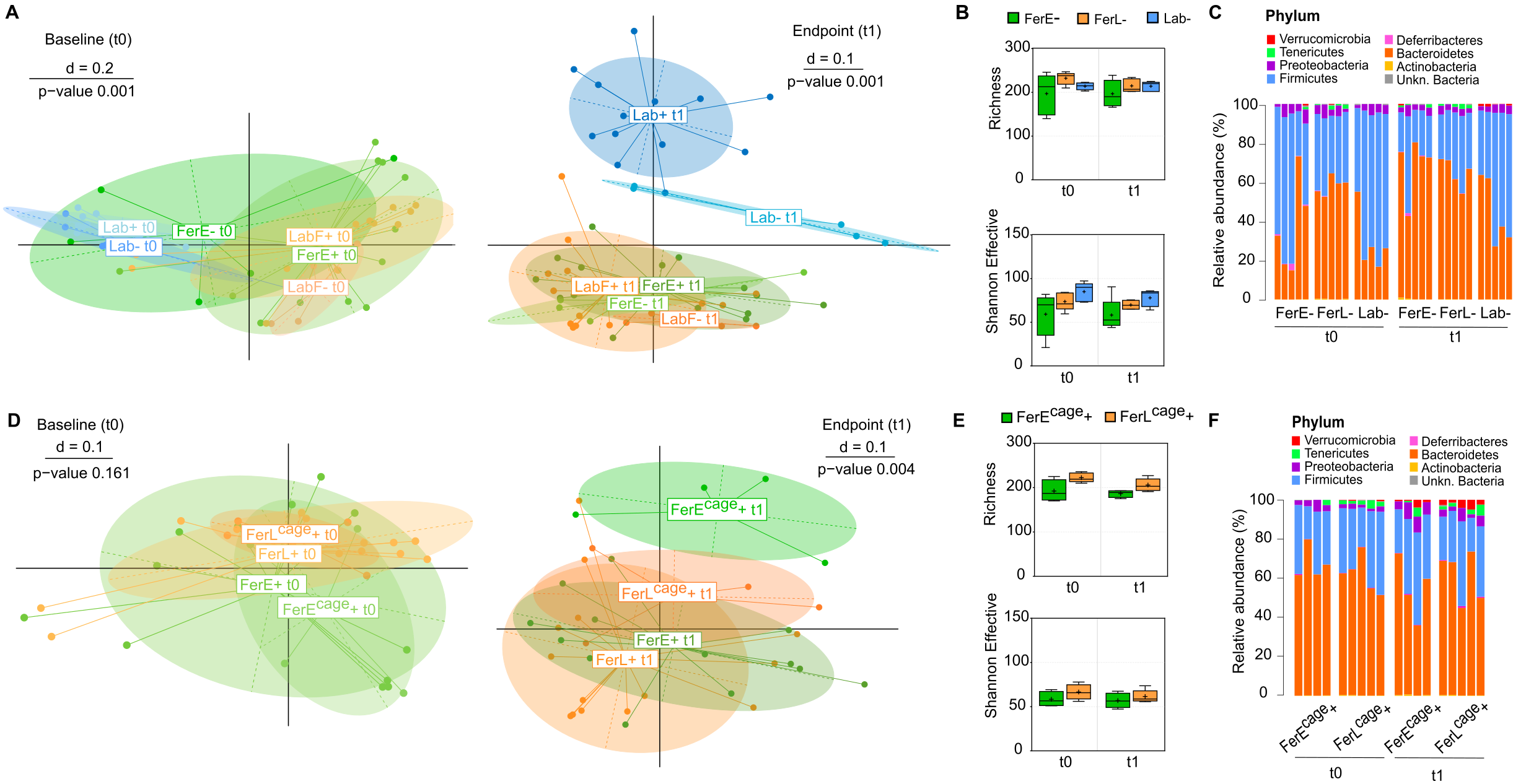

Supplement: Supplemental Material [file KGMI_A_1993581_SM4446.zip › SupplementaryFigureS6_rev1_2.tiff]

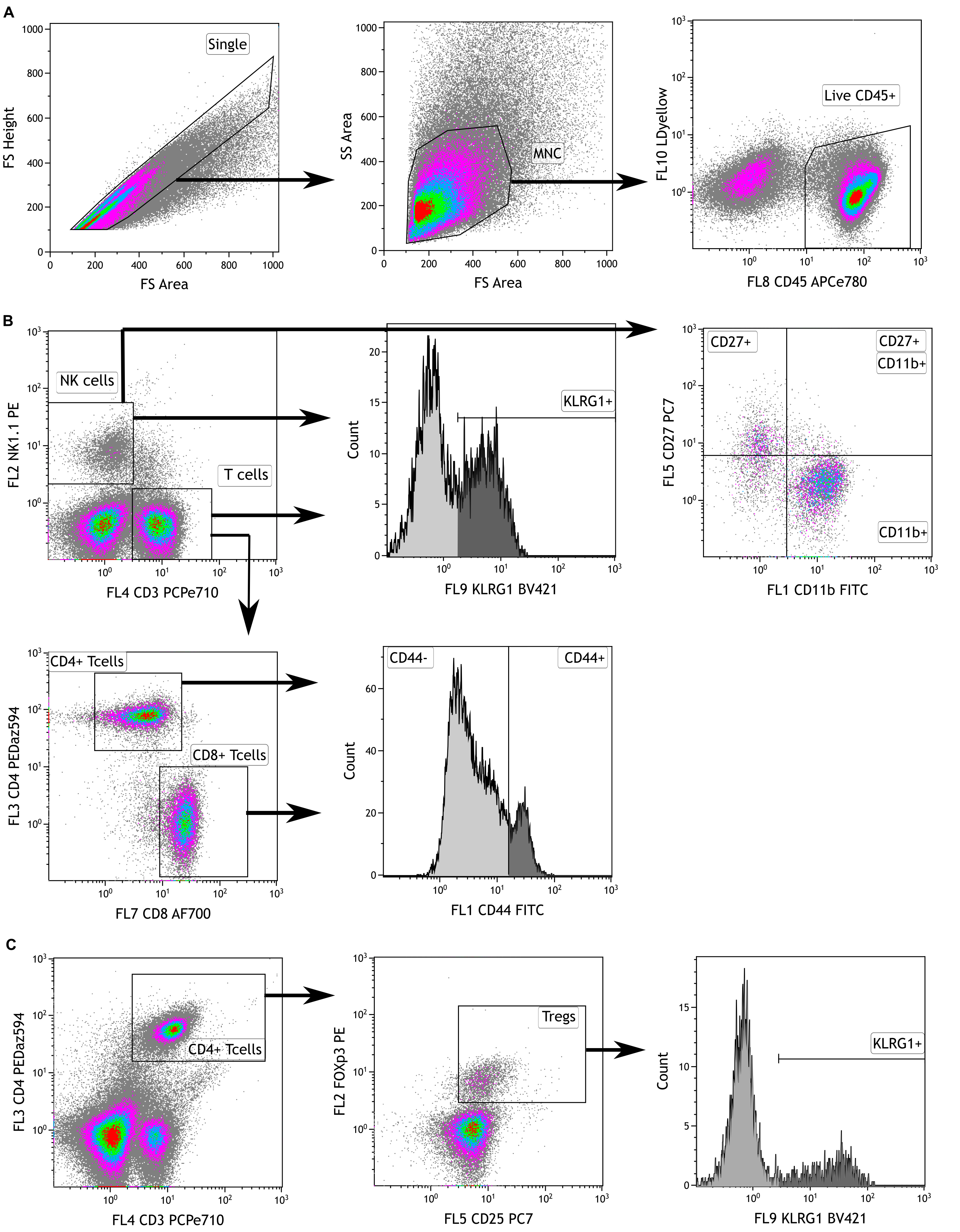

Supplement: Supplemental Material [file KGMI_A_1993581_SM4446.zip › SupplementaryFigureS7.tiff]

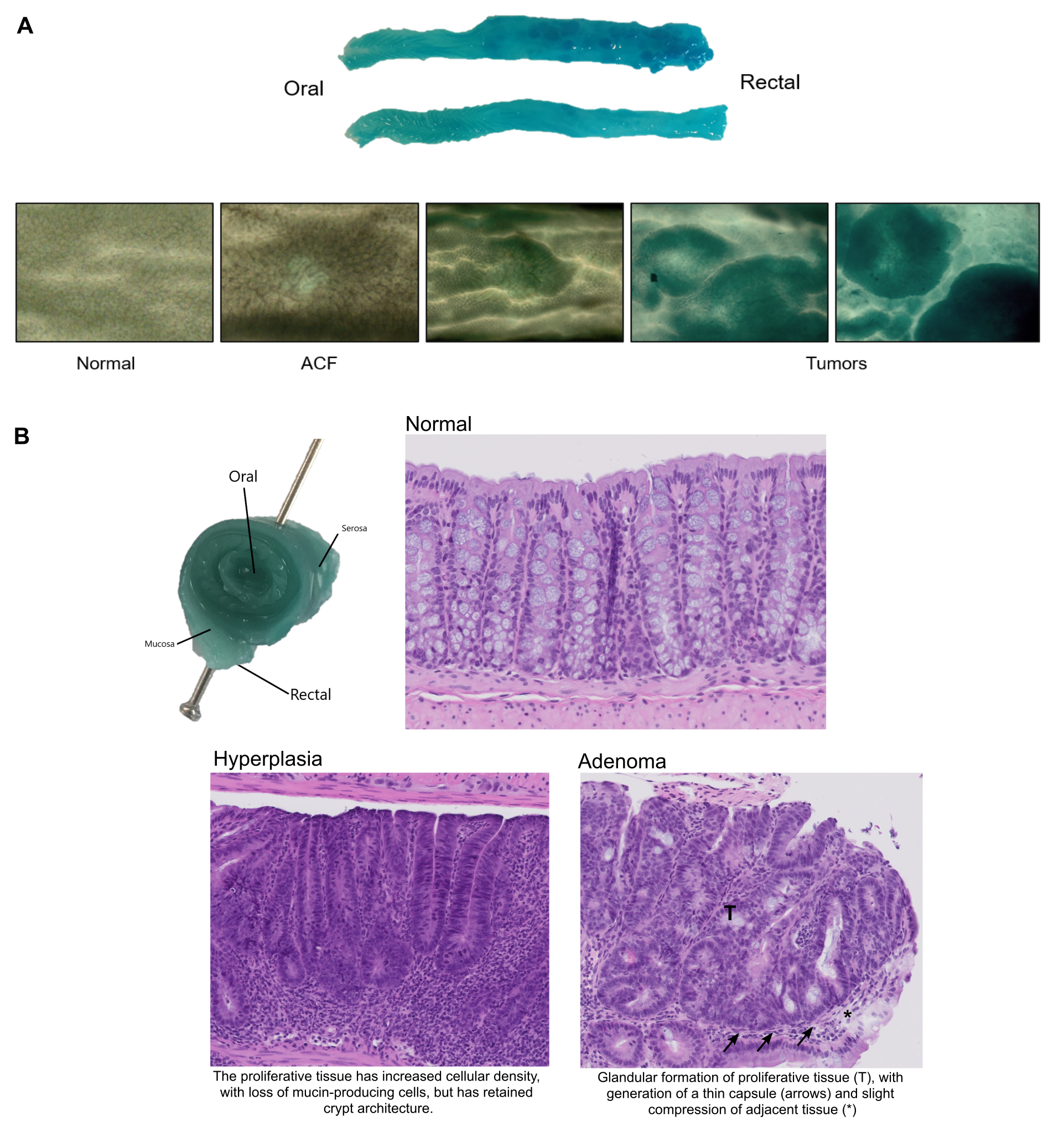

Supplement: Supplemental Material [file KGMI_A_1993581_SM4446.zip › SupplementaryFigureS8_rev1.tiff]
